# Repetitive Transcranial Magnetic Stimulation for Treatment of Autism Spectrum Disorder: A Systematic Review and Meta-Analysis

**DOI:** 10.3389/fnint.2018.00027

**Published:** 2018-07-09

**Authors:** J. Bernardo Barahona-Corrêa, Ana Velosa, Ana Chainho, Ricardo Lopes, Albino J. Oliveira-Maia

**Affiliations:** ^1^Champalimaud Clinical Centre, Champalimaud Centre for the Unknown, Lisbon, Portugal; ^2^Champalimaud Research, Champalimaud Centre for the Unknown, Lisbon, Portugal; ^3^Department of Psychiatry and Mental Health, Centro Hospitalar de Lisboa Ocidental, Lisbon, Portugal; ^4^NOVA Medical School|Faculdade de Ciências Médicas, Universidade Nova de Lisboa, Lisbon, Portugal; ^5^CADIN–Neurodesenvolvimento e Inclusão, Cascais, Portugal; ^6^Centro de Investigação e de Intervenção Social (CIS-IUL), Instituto Universitário de Lisboa (ISCTE-IUL), Lisbon, Portugal

**Keywords:** autism, Asperger's, non-invasive brain stimulation, TMS, rTMS, TBS, meta-analysis

## Abstract

**Background:** Autism Spectrum Disorder (ASD) is a neurodevelopmental disorder manifesting as lifelong deficits in social communication and interaction, as well as restricted repetitive behaviors, interests and activities. While there are no specific pharmacological or other physical treatments for autism, in recent years repetitive Transcranial Magnetic Stimulation (rTMS), a technique for non-invasive neuromodulation, has attracted interest due to potential therapeutic value. Here we report the results of a systematic literature review and meta-analysis on the use of rTMS to treat ASD.

**Methods:** We performed a systematic literature search on PubMed, Web of Science, Science Direct, Bielefeld Academic Search, and Educational Resources Information Clearinghouse. Search terms reflected diagnoses and treatment modalities of interest. Studies reporting use of rTMS to treat core ASD or cognitive symptoms in ASD were eligible. Two researchers performed article selection and data extraction independently, according to PRISMA guidelines. Changes in ASD clinical scores or in cognitive performance were the main outcomes. Random effects meta-analysis models were performed.

**Results:** We found 23 eligible reports, comprising 4 case-reports, 7 non-controlled clinical trials, and 12 controlled clinical trials, comparing the effects of real TMS with waiting-list controls (*n* = 6) or sham-treatment (*n* = 6). Meta-analyses showed a significant, but moderate, effect on repetitive and stereotyped behaviors, social behavior, and number of errors in executive function tasks, but not other outcomes. Most studies had a moderate to high risk of bias, mostly due to lack of subject- and evaluator-blinding to treatment allocation. Only 5 studies reported stability of these gains for periods of up 6 months, with descriptions that improvements were sustained over time.

**Conclusions:** Existing evidence supports that TMS could be useful to treat some dimensions of ASD. However, such evidence must be regarded with care, as most studies did not adequately control for placebo effects. Moreover, little is known regarding the most effective stimulation parameters, targets, and schedules. There is an urgent need for further randomized, double-blind, sham-controlled trials, with adequate follow-up periods, to test the efficacy of transcranial magnetic stimulation to treat these disorders. Available evidence must be regarded as preliminary and insufficient, at present, to support offering TMS to treat ASD.

## Introduction

### Rationale

Autism Spectrum Disorder (ASD) is a diagnostic category that encompasses a group of neurodevelopmental disorders with varying degrees of severity, sharing a common syndromatic core dyad of significant deficits in social communication and interaction, and repetitive behaviors with restricted interests and activities (American Psychiatric Association, 2013; Volkmar and McPartland, 2014). Deficits in communication can present as absent or severely impaired language development, and restricted interests can vary from extremely stereotyped, non-functional repetitive behavior, to milder forms of over-involvement or over-investment in a limited span of interests and activities (Volkmar and McPartland, 2014). Other features that are frequently associated include cognitive delay, epilepsy, and motor clumsiness, as well as significant psychiatric co-morbidity, such as obsessive-compulsive disorder, affective disorders, and attention deficit and hyperactivity disorder (Stahlberg et al., 2004; Russell et al., 2005; Hutton et al., 2008; Mouridsen et al., 2008). Furthermore, ASD patients are impaired in a number of neuropsychological tasks, namely theory of mind tests and tasks depending on executive functions such as working memory, prepotent response inhibition or interference control (Kana et al., 2015; Wallace et al., 2015; Geurts and Lever, 2017). ASD is a lifelong disorder, and most patients remain severely impaired in terms of psychosocial functioning throughout their adult lives (Billstedt et al., 2005; Farley et al., 2009). Currently there are important limitations in the treatment of ASD. Pharmacological treatment is only indicated for psychiatric comorbidity and has no measurable impact on ASD core manifestations. Available non-pharmacological interventions, on the other hand, are generally expensive, time-consuming and have modest results (Buescher et al., 2014).

In more recent years the availability of non-invasive neuromodulation techniques has raised hope that they might prove an effective tool in the treatment of ASD core manifestations (Oberman and Enticott, 2015). Transcranial Magnetic Stimulation (TMS) is a non-invasive neuromodulation technique that has attracted particular interest. TMS uses electromagnetic induction to generate transient, localized electrical fields in the brain cortex, causing depolarization and firing of local neurons (Hallett, 2000). Repetitive TMS (rTMS) delivers patterns of multiple TMS pulses over a chosen brain area, at frequencies that typically vary between 0.5 Hz and 20 Hz (Pascual-Leone et al., 1999). At low frequencies, rTMS results in long-term suppression of cortical excitability on the target cortical tissue, while at frequencies above 5 Hz, rTMS mostly induces long-term facilitation of cortical excitability (Pascual-Leone et al., 1998). These effects, however, are subject to significant inter-individual variability (Maeda et al., 2000). An alternative modality of rTMS delivery is Theta-burst Stimulation (TBS), where TMS pulses are delivered in 50 Hz 3-pulse bursts, at 200 ms intervals (i.e., with a 5 Hz burst frequency). Continuous TBS (cTBS) suppresses cortical excitability, while intermittent TBS (iTBS), where TBS is delivered for 2 s every 10 s, has facilitatory effects on the cortex (Huang et al., 2005).

Use of TMS in humans is generally safe and well tolerated, and rTMS has received formal approval for the treatment of drug-resistant Major Depression (Rossi et al., 2009). For these reasons, TMS has become an attractive treatment option in ASD, stimulating interest in particular among ASD patients and their parents, who are characteristically attentive to innovative treatments (Levy and Hyman, 2005). Unfortunately, it remains unsettled whether there is indeed a place for rTMS in the treatment arsenal for ASD. In a narrative review published in 2016, Oberman et al. (2016) found 12 studies and three case-reports describing the use of rTMS protocols in ASD with therapeutic intent (Oberman et al., 2016). Stimulation parameters and targets varied widely across studies, and most of them were open-label. The authors concluded that evidence for improvement of specific ASD-related behavioral symptoms resulting from rTMS applied to specific cortical regions, while encouraging, was limited. In fact, results at the individual level were mixed, and large-scale controlled trials were lacking. Here we describe an update of this work, 3 years after its initial online publication. In further development of prior methods (Oberman et al., 2016) we conducted a systematic review of the literature, for published reports on the therapeutic use of rTMS in patients with ASD. Furthermore, we performed meta-analyses of the data in these reports, thus offering the first estimation of pooled effects sizes for the effects of TMS on several clinical dimensions of ASD.

### Objectives

While there are clinical programs offering rTMS to treat ASD symptoms, the evidence-base sustaining efficacy and safety in this context remains unclear. Moreover, the optimal stimulation parameters and brain targets remain undefined. Here we conducted a systematic literature review and meta-analysis to explore available data regarding the therapeutic use of rTMS for ASD.

### Research question

We were interested in understanding whether the published literature supports use of TMS for amelioration of core-symptoms of ASD, or of the cognitive impairments associated with these disorders.

## Methods

### Study design

We conducted a systematic review of the literature with meta-analyses of studies reporting quantitative data, separated according to the outcome of interest.

### Participants, interventions, comparators

We were interested in reviewing studies conducted in patients with any ASD, regardless of age or gender. Given the limitations in the available literature, we were interested in any clinical trial, regardless of design, as well as case-studies or case-series, reporting the effects of rTMS on ASD core symptoms and/or cognitive performance.

### Systematic review protocol

The systematic review was conducted according to a protocol following PRISMA guidelines.

### Search strategy

The search was performed on PubMed, Web of Science, BASE (Bielefeld Academic Search), ERIC (educational resources information clearinghouse) and Science Direct, between June 2016 and January 2018. Search terms reflected the diagnoses of interest (Autism, Autism Spectrum Disorders, Asperger) and the interventions of interest (Transcranial, TMS, rTMS, TBS).

After eliminating duplicates, two researchers reviewed the list of articles separately, selecting eligible reports according to PRISMA procedures. Articles in English, French, German, Portuguese or Spanish were considered, regardless of publication date or country of origin. Only full articles published in peer-review journals were considered. Literature reviews were excluded, but were screened for additional references, as were reference lists of eligible articles.

### Data sources, studies sections, and data extraction

Two researchers extracted data separately according to PRISMA guidelines. From each eligible paper, whenever possible we extracted information separately for patients included in the active treatment and those in the control intervention arm, namely author, publication year and journal, number of participants, gender distribution, mean age, mean intelligence quotient, number of dropouts, type of TMS stimulation, stimulation parameters and target, stimulation schedule, behavioral outcome measures, cognitive outcome measures, number and nature of any reported side-effects, follow-up period duration, additional information. Risk of selection bias, performance bias, attrition bias and detection bias were also registered according to Cochrane guidelines (Higgins and Green, 2006).

For each outcome variable from each eligible study, depending on what was reported in the original paper, we extracted pre-treatment and post-treatment mean and standard deviation, *F*-value from Analysis of Variance tests, and/or Cohen's d estimates. If reported we also extracted mean change from baseline, standard deviation of this measure and/or paired *t*-test *t*-values. Data were extracted either directly from the text and tables or extrapolated from figures. In the latter situation values (mean and standard deviation or standard error of the mean) were extracted using Adobe Acrobat Reader measurement tools. To account for measurement error, each value from each figure was measured five times, and the mean value computed. In cases where data included in the original manuscript were insufficient, we contacted the corresponding author to request further information. Data is described in the manuscript as mean ± standard deviation. Original data supporting the conclusions of this manuscript will be made available by the authors, without undue reservation, to any qualified researcher.

### Data analysis

To estimate effect-sizes we computed Hedges g for each study, using Meta-essentials workbooks (http://www.erim.eur.nl/research%7D-support/meta-essentials/). For controlled studies, computations were conducted using one of the following, according to what was reported: post-treatment means and standard deviations of both the active treatment and the control intervention groups, *F*-values from Analysis of Variance tests, or Cohen's d estimates. When, instead of raw outcome data, authors reported mean change from baseline, within-group standard deviation for the outcome measures was estimated using $\sigma =\frac{\sigma D}{\sqrt{2\left(1-\rho \right)}}$, where σ*D* stands for the standard deviation of the difference relative to baseline, and ρ for the correlation between pre- and post-treatment scores (Borenstein et al., 2009a). For non-controlled studies a repeated measures approach was adopted for calculation of Hedges g, with use of either pre- and post-treatment means and standard deviations, or mean change from baseline, the respective standard deviation ($SD_{D}^{2}$) and correlation coefficient between pre- and post-treatment scores. When the latter was not provided we computed the correlation coefficient using the formulas $r=\;\frac{\;SDpre^{2}\;+\;SDpost^{2}\;-SD_{D}^{2}\;}{2\;x\;SDpre\;x\;SDpost}$ and $SD_{D}^{2}=\frac{{n\left(Mpost-Mpre\right)}^{2}}{t_{RM}^{2}}$, where $t_{RM}^{2}$ is the pre-post paired *t*-test value, *Mpre* is the pre-TMS mean score and *Mpost* the post-TMS mean score (Morris and DeShon, 2002). When reported data were insufficient for this computation, *r*-values were extrapolated from other papers with similar clinical populations, or, when this was not possible, from published data on the test-retest reliability of that specific measure.

Where a sufficient number of studies were available, results for relevant symptom dimensions or cognitive functions were included in random effects model meta-analyses, separately for controlled and uncontrolled studies. Controlled studies that did not report outcome data for the control group regarding a specific clinical outcome dimension were entered in the corresponding meta-analysis of uncontrolled studies. For each meta-analysis, the decision to include any given study was essentially determined by the availability of sufficient quantitative data reporting treatment effects on the clinical dimension of interest. Due to the small number of available studies, study quality was not considered in the decision of inclusion. Meta-essentials workbooks 1, 3, and 4 were used to compute bias-adjusted standardized mean differences (Hedges g, expressed as 95% confidence intervals−95% CI), as well as combined effect sizes with hypothesis testing. Individual studies were weighed according to the inverse variance weighting method, with an added between-studies variance component based on the DerSimonian-Laird estimator (Sánchez-Meca and Marín-Martínez, 2008). Confidence intervals were estimated using the weighted variance method, as described previously (Sánchez-Meca and Marín-Martínez, 2008). This approach takes into account the uncertainty resulting from the need to estimate heterogeneity variance and within-study variances, resulting in wider estimated confidence intervals for the combined effect size in analyses based on small numbers of studies. In the latter situation, and especially when heterogeneity is high, confidence intervals may include 0 even when classical z-distribution confidence intervals would not. To assess heterogeneity of studies, in each meta-analysis we used Cochrane's *Q-*test to examine the null hypothesis that all studies estimated the same effect. We further computed I^2^ to estimate the ratio of true heterogeneity to total observed variation, and Tau^2^ (T^2^) to estimate between-study variance (Higgins et al., 2003). Publication bias was examined by means of funnel-plots, with Egger regression and trim-and-fill analysis for estimation of the adjusted effect size and of missing studies (Borenstein et al., 2009b).

## Results

### Study selection and characteristics

**Figure 1:**
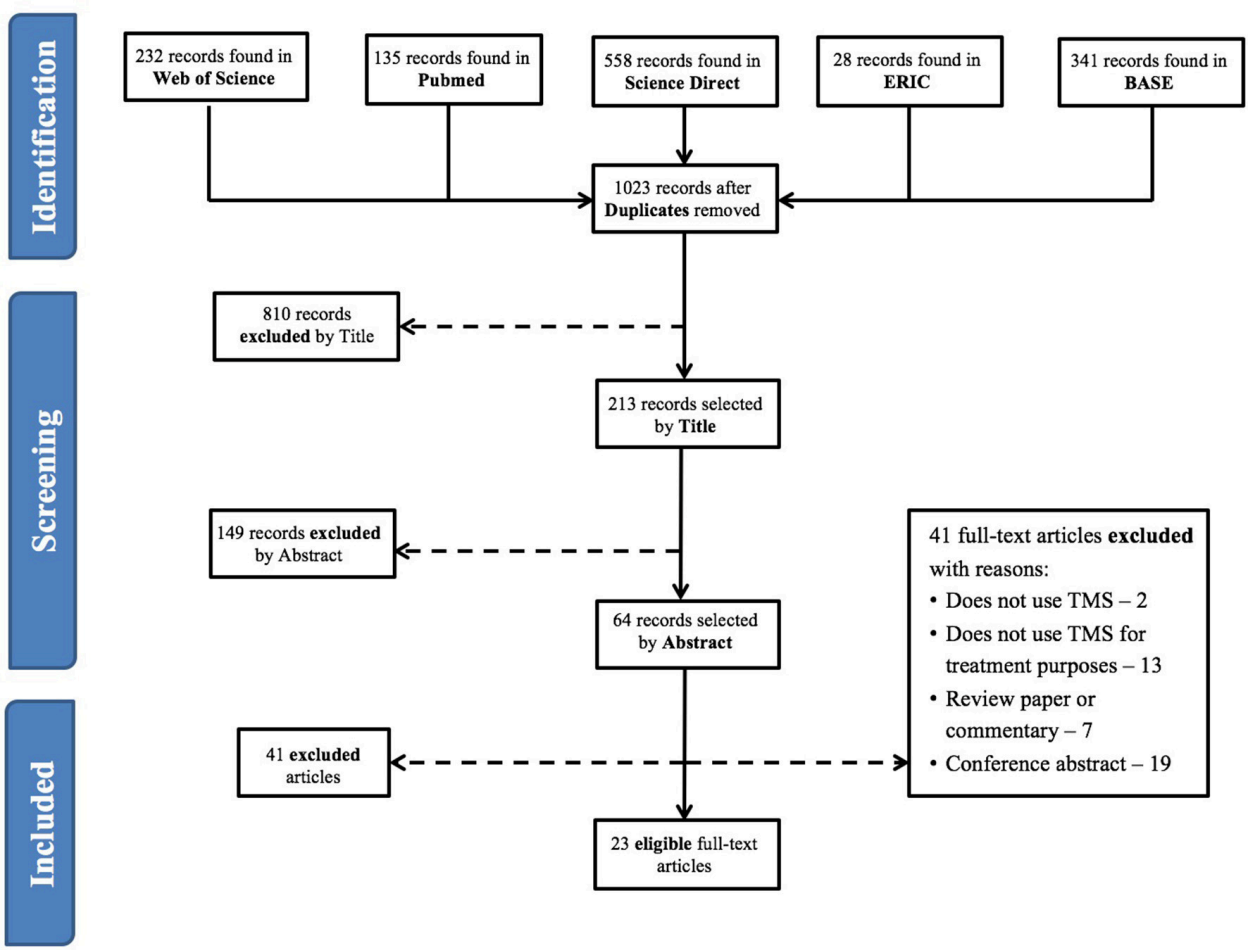
Article selection flowchart (according to PRISMA Statement).

Figure 1 shows a flow diagram of the process leading to the identification of 23 eligible papers, using rTMS for therapeutic purposes in subjects with ASD (refer to Tables 1–3 for individual study details). Four of these papers were case-reports (Enticott et al., 2011; Niederhofer, 2012; Cristancho et al., 2014; Avirame et al., 2017), 7 were non-controlled trials (Sokhadze et al., 2010, 2016, 2017; Casanova et al., 2014; Wang et al., 2016; Abujadi et al., 2017; Gómez et al., 2017) and the remaining 12 were controlled trials (Sokhadze et al., 2009, 2012, 2014a,b; Baruth et al., 2010; Fecteau et al., 2011; Casanova et al., 2012; Enticott et al., 2012, 2014; Panerai et al., 2014; Anninos et al., 2016; Ni et al., 2017). Six of the controlled studies used sham rTMS as the control intervention (Fecteau et al., 2011; Enticott et al., 2012, 2014; Panerai et al., 2014; Anninos et al., 2016; Ni et al., 2017) while the remaining 6 compared rTMS-treated patients with wait-list controls. Three studies recruited exclusively adult subjects to the experimental arm (Fecteau et al., 2011; Enticott et al., 2014; Ni et al., 2017), with the remainder focusing predominantly on children and adolescents. Overall, data were reported from 371 rTMS-treated patients, with a mean age of 15.9 ± 5.2 years, 5 of whom in case-reports (26.4 ± 9.3 years-old) and the remaining 366 from intervention studies (15.8 ± 5.1 years-old). The overwhelming majority of studies recruited patients with an IQ higher than 80. The 4 trials reported in the paper by Panerai et al. (2014) were all performed on subjects with severe cognitive impairment, while Abujadi et al. (2017), Baruth et al. (2010), and Wang et al. (2016) included patients both with and without cognitive impairment.

**Table 1:** Summary table of study design, modality of TMS, recruited subjects, and study quality.

**Source**	**TMS modality**	**Experimental group**			**Control group**			**Control intervention**	**Risk of selection Bias**	**Risk of performance Bias**	**Risk of attrition Bias**	**Risk of detection Bias**
		***N***	**Mean age (years)**	**Intellectual ability**	***N***	**Mean age (years)**	**Intellectual ability**					
Anninos et al., 2016	Pico tesla TMS	10 (6 male)	8.3 ± 2.1	Not reported	Same group subject to sham TMS				Low (randomized order)	Low (double-blind)	Low (no dropouts)	Low (double-blind)
Baruth et al., 2010	rTMS	16	13.9 ± 5.3	IQ = 86 ± 24.7	9 ASD + 20 healthy subjects	ASD: 13.5 ± 2; healthy subjects: 15.3 ± 5.1	Not reported	Waiting List	Low (randomized)	High (unblinded)	Low (no dropouts)	High (unblinded)
Casanova et al., 2012	rTMS	25	12.9 ± 3.1	IQ>80	20	13.1 ± 2.2	IQ>80	Waiting List	Low (randomized)	High (unblinded)	Low (no dropouts)	High (unblinded)
Enticott et al., 2012	rTMS	11 (10 male)	17.55 ± 4.06	Not reported	Same group subject to sham TMS			Sham rTMS (left primary motor cortex)	Low (randomized order)	High (unblinded)	Low (no dropouts)	High (unblinded)
Enticott et al., 2014	Deep rTMS	15 (13 male)	33.87 ± 13.07	Not reported	13 adults (10 males)	30.54 ± 9.83	Not reported	Sham rTMS	Low (randomized)	Low (double-blind)	Moderate (2 dropouts)	Low (evaluator-blinded)
Fecteau et al., 2011	rTMS	10	36.6 ± 16.0	IQ = 122.4 ± 7.2	10 healthy controls; sham rTMS in experimental subjects			Sham rTMS (central lobe midline)	Low (pseudo-randomized, multiple crossover)	Low (double-blind)	Low (no dropouts)	Low (double-blind)
Ni et al., 2017	iTBS	19 (14 male)	20.8 ± 1.4	IQ = 100.5 ± 14	Same group subject to sham iTBS			Sham iTBS (over the inion)	Low (randomized order)	High (unblinded)	Low (no dropouts)	High (unblinded)
Panerai et al., 2014; Study I	rTMS	9	13.56 ± 1.83	Severe mental retardation	Same group subject to sham TMS			Sham TMS	Low (randomized order)	Low (double-blind)	Low (no dropouts)	Low (double-blind)
Panerai et al., 2014; Study II	rTMS	HFrTMS: 6; LFrTMS: 6	HFrTMS: 13.7 ± 1.96; LFrTMS: 13.33 ± 1.88	Severe mental retardation	5	13.24 ± 2.95	Severe mental retardation	Sham TMS	Low (randomized)	Low (double-blind)	Low (no dropouts)	Low (double-blind)
Panerai et al., 2014; Study III	rTMS	6	16.13 ± 3.11	Severe mental retardation	Same group subject to sham TMS			Sham TMS	Low (randomized order)	Low (double-blind)	High (2 dropouts)	Low (double-blind)
Panerai et al., 2014; Study IV	rTMS	HFrTMS: 4; HFrTMS+ Eye–hand integration training: 4	HFrTMS: 12.79 ± 2.88; HFrTMS+training 13.75 ± 5.18	Severe mental retardation	5	14.17 ± 4.24	Not reported	Eye–hand integration training	Low (randomized)	High (unblinded)	Low (no dropouts)	High (unblinded)
Sokhadze et al., 2009	rTMS	8	18.3 ± 4.8	IQ = 104 ± 19.9	5	16.2 ± 5.7	IQ: 100.8 ± 12.4	Waiting List	High (not randomized)	High (unblinded)	Low (no dropouts)	High (unblinded)
Sokhadze et al., 2012	rTMS	20 (16 male)	13.5 ± 2.5	IQ = 90.8 ± 15.2	20 (16 male)	13.5 ± 2.5	Not reported	Waiting List	High (no concealment, unrandomized, unpaired)	High (unblinded)	Low (no dropouts)	High (unblinded)
Sokhadze et al., 2014b	rTMS	20	14.7 ± 3.3	IQ>80	22	14.2 ± 2.8	IQ>80	Waiting List	High (no concealment, unrandomized, unpaired)	High (unblinded)	Low (no dropouts)	High (unblinded)
Sokhadze et al., 2014a	rTMS	27	14.8 ± 3.2	IQ>80	27	14.1 ± 2.6	IQ>80	Waiting List	High (no concealment, unrandomized, unpaired	High (unblinded)	Low (no dropouts)	High (unblinded)
Sokhadze et al., 2016	rTMS	25 (19 male)	13.6 ± 3.22	IQ>80	21 healthy controls	14.9 ± 4.3	Not reported	No control intervention	Not applicable	High (unblinded)	Moderate (2 dropouts)	High (unblinded)
Abujadi et al., 2017	iTBS	10 (male)	9-17	IQ>50	No control group				Not applicable	High (open label)	Low (no dropouts)	High (open label)
Casanova et al., 2014	rTMS	18 (14 male)	13.1 ± 2.2	IQ>80	No control group				Not applicable	High (unblinded)	High (2 dropouts; 2 excluded)	High (unblinded)
Gómez et al., 2017	rTMS/tDCS	24	12.2 (rTMS≥11;tDCS < 11)	Not reported	No control group				Not applicable	High (unblinded)	Low (no dropouts)	High (unblinded)
Sokhadze et al., 2010	rTMS	13 (12 male)	15.6 ± 5.8	IQ = 94.3 ± 16.6	No control group				Not applicable	High (unblinded)	Moderate (1 dropout)	High (unblinded)
Sokhadze et al., 2017	rTMS	32 enrolled; final sample 27 (21 male)	12.52 ± 2.85	IQ>80	No control group				Not applicable	High (unblinded)	High (3 droputs; 2 cases excluded)	High (unblinded)
Wang et al., 2016	rTMS	33 (28 male)	12.88 ± 3.76	IQ>80; 10 had IQ 65-79	No control group				Not applicable	High (unblinded)	Moderate (3 dropouts)	High (unblinded)
Avirame et al., 2017, case 1	Deep TMS	1 (female)	25	Not reported	No control group				Not applicable	Not applicable	Not applicable	Not applicable
Avirame et al., 2017, case 2	Deep TMS	1 (male)	30	Not reported	No control group				Not applicable	Not applicable	Not applicable	Not applicable
Cristancho et al., 2014	TMS	1 (male)	15	Not reported	No control group				Not applicable	Not applicable	Not applicable	Not applicable
Enticott et al., 2011	Deep rTMS	1 (female)	20	Not reported	Not reported				Not applicable	Not applicable	Not applicable	Not applicable
Niederhofer, 2012	rTMS	1 (female)	42	Not reported	Same patient subject to sham TMS				Not applicable	Not applicable	Not applicable	Not applicable

*ASD, Autism Spectrum Disorder; HFrTMS, High-frequency repetitive transcranial magnetic stimulation; iTBS, Intermittent Theta-burst stimulation; LFrTMS, Low-frequency repetitive transcranial magnetic stimulation; rTMS, Repetitive transcranial magnetic stimulation; tDCS, Transcranial direct current stimulation; TMS, Transcranial magnetic stimulation*.

One study used a pico-Tesla TMS protocol (Anninos et al., 2016) while two others used TBS protocols (Abujadi et al., 2017; Ni et al., 2017). Pico-Tesla TMS is a technology invented and used by a specific research group (Anninos and Tsagas, 1995). It uses a modified helmet containing up to 122 coils arranged in several arrays. This allows for delivery of low-intensity transcranial magnetic stimulation, at the pico-Tesla level (1 pico-Tesla = 10^−12^ Tesla), simultaneously to several brain areas, at frequencies of 2–7 Hz (Anninos et al., 2016). The remaining studies used other, more conventional rTMS methods. Most studies targeted stimulation to the dorsolateral prefrontal cortex (DLPFC), either bilaterally or in the left hemisphere, using low stimulation frequencies (0.5–1 Hz). Ni et al. (2017) additionally stimulated the posterior superior temporal sulcus using iTBS, while Fecteau et al. (2011) used 1 Hz rTMS to stimulate the pars triangularis and pars opercularis of the inferior frontal gyrus, bilaterally. Avirame et al. (2017) and Enticott et al. (2011, 2014) targeted the dorsal medial prefrontal cortex (DMPFC) with stimulation frequencies of 5 Hz. Panerai et al. (2014) stimulated the left premotor cortex (and in a subgroup of patients also the right premotor cortex) at 8 Hz, while Enticott et al. (2012) chose to stimulate the left primary motor cortex and supplementary motor area with 1 Hz rTMS. The pico-Tesla TMS trial targeted stimulation to the vertex, frontal and occipital cortex, left and right parietal cortex, and left and right temporal cortex, at frequencies of 8–13 Hz (Anninos et al., 2016). Treatment schedules also varied widely. While most studies delivered treatment in weekly or twice-weekly sessions, some delivered daily sessions for 5–29 days consecutively (Niederhofer, 2012; Cristancho et al., 2014; Enticott et al., 2014; Panerai et al., 2014; Anninos et al., 2016; Avirame et al., 2017; Gómez et al., 2017). A limited number of studies used a single session in a proof of concept approach (Fecteau et al., 2011; Panerai et al., 2014; Ni et al., 2017). Only two studies used neuronavigation to guide stimulation of the intended cortical region. All studies used conventional figure of eight coils, with the exception of Enticott et al. (2014), where an H-coil was used, and Anninos et al. (2016), where a specially designed helmet, containing up to 122 coils, was chosen.

### Synthesized findings and risk of bias

#### Effects on behavior

Sixteen studies assessed the effects of rTMS on ASD symptoms or ASD associated behavioral problems. Seven of these studies were prospective and non-controlled (Sokhadze et al., 2010, 2016, 2017; Casanova et al., 2014; Wang et al., 2016; Abujadi et al., 2017; Gómez et al., 2017). Data from the study by Gómez et al. (2017), who used both rTMS and transcranial direct current stimulation in their sample, could not be used as authors did not provide separate outcome data for the rTMS intervention. Two other studies, while reporting a waiting-list control design, did not provide data for the control group, and were thus analyzed jointly with non-controlled studies in a pre-post meta-analysis design (Sokhadze et al., 2009; Baruth et al., 2010). The remaining seven studies were controlled with either waiting-list controls (Casanova et al., 2012; Sokhadze et al., 2014a,b), sham-rTMS (Enticott et al., 2014; Ni et al., 2017), or sham pico-Tesla TMS (Anninos et al., 2016, 2017). Anninos et al. (2016) provided only a qualitative description of symptom change in their treated patients, with no quantitative assessment of change. In a recently published study from the same research group, similarly-designed to test the effects of pico-Tesla TMS on ASD symptoms, and that was not retrieved by our systematic search strategy (Anninos et al., 2017), clinical improvement was reported in 6 of 8 children, but described only qualitatively, rather than quantitavely, and was also not included in the data synthesis. The different studies assessed the effects of rTMS on one or more of three behavioral dimensions: restricted and repetitive or stereotyped behavior; impairments in social behavior; other non-core ASD-related symptoms, such as hyperactivity or irritability. The specific instruments used in each study are detailed in Table 3.

**Figure 2:**
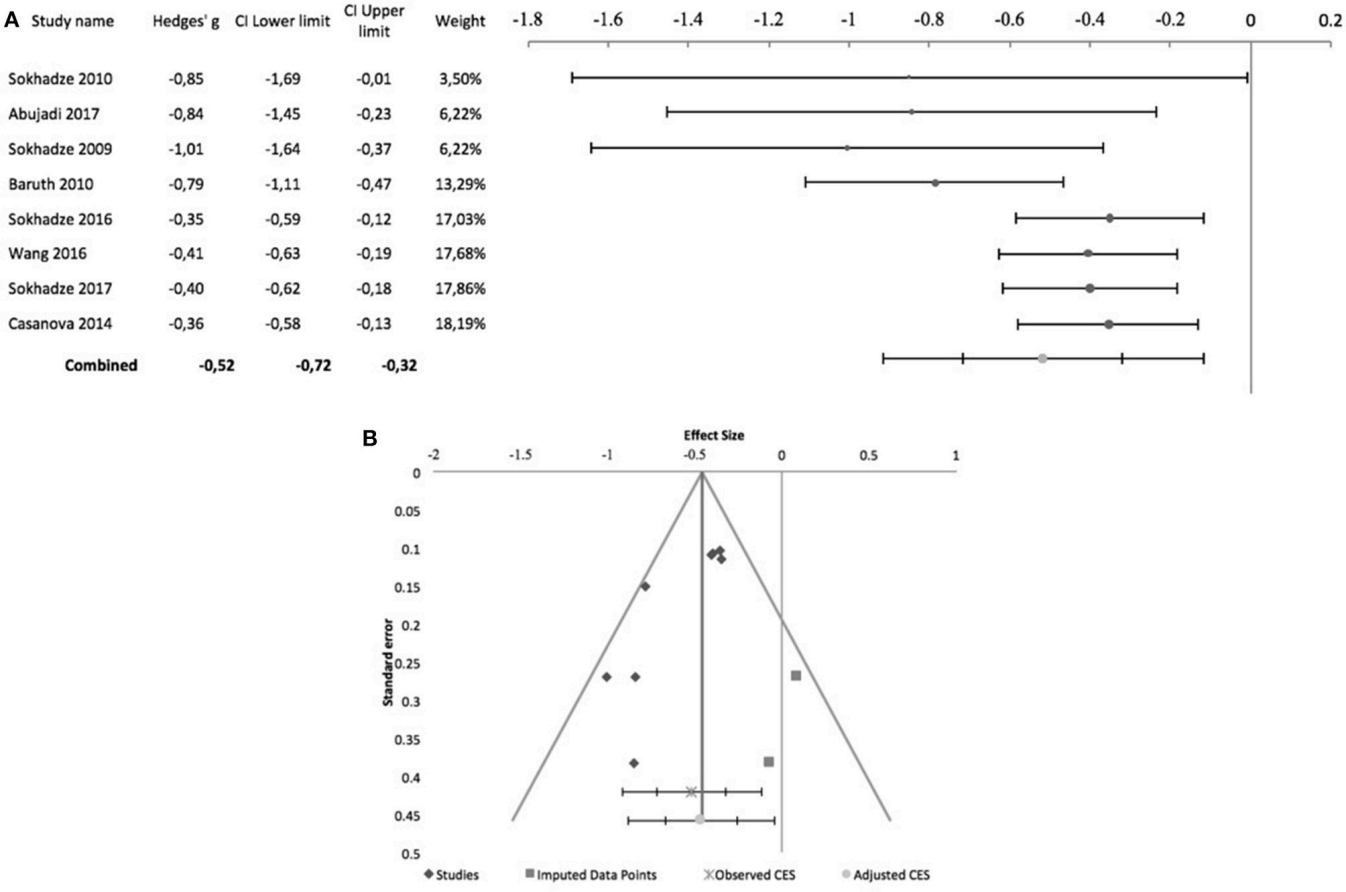
Effects of rTMS on repetitive and restricted behavior in non-controlled studies. **(A)** Forest-plot. Dots represent each study, with dot size reflecting study weight in the model and error bars indicating the effect size (with confidence interval). The lower line represents the combined effect size with its confidence interval (narrow interval) and its 95% prediction interval (wide interval). The latter gives the range in which, in 95% of the cases, the outcome of a future study will fall, assuming that the effect sizes of studies are normally distributed. **(B)** Funnel-plot. There is evidence of significant publication bias. Square-shaped dots represent studies imputed by trim-and-fill analysis.

With regards to stereotyped and repetitive behavior, the effects of rTMS were measured using the total Repetitive Behavior Scale-Revised (RBS-R), stereotypy subscale of the Aberrant Behavior Checklist (ABC), Attention Switching subscale of the Autism Spectrum Quotient, or compulsion subscale of the Yale-Brown Obsessive-Compulsive Scale. In all of these scales higher scores reflect higher symptom severity. Figure 2 shows the forest plot of the pre-post analysis for 8 non-controlled studies. Three of these studies included a control group but were included in this meta-analysis as no outcome data were reported for the control group (Sokhadze et al., 2009, 2016; Baruth et al., 2010). All of the eight studies incurred in risk of bias, as none was subject- or rater blinded, and none used an intent-to-treat approach regarding dropouts. All but Abujadi et al. (2017), that used iTBS, applied low-frequency rTMS with stimulus frequencies between 0.5 and 1 Hz. Sokhadze et al. (2010) and Sokhadze et al. (2009) targeted the left DLPFC, while Abujadi et al. (2017) stimulated the right DLPFC. The remaining five trials chose bilateral stimulation of the DLPFC. We found a significant combined effect size of−0.52 (95% CI: −0.72 to −0.32; *z* = −6.17; *p* < 0.001), with a significant Cochrane's *Q-*test of Heterogeneity (*Q* = 14.4, *p* = 0.04), *I*^2^-value of 51.5% and T^2^ of 0.02. The funnel plot and trim-and-fill analysis were suggestive of publication bias, with two missing negative studies and an adjusted effect size of−0.46 (95% CI: −0.67 to −0.25; Figure 2). Egger statistic was also significant (intercept = −3.2, 95%CI: −5.5 to −0.9; *t* = −3.3, *p* = 0.02), suggesting a significant bias.

**Figure 3:**
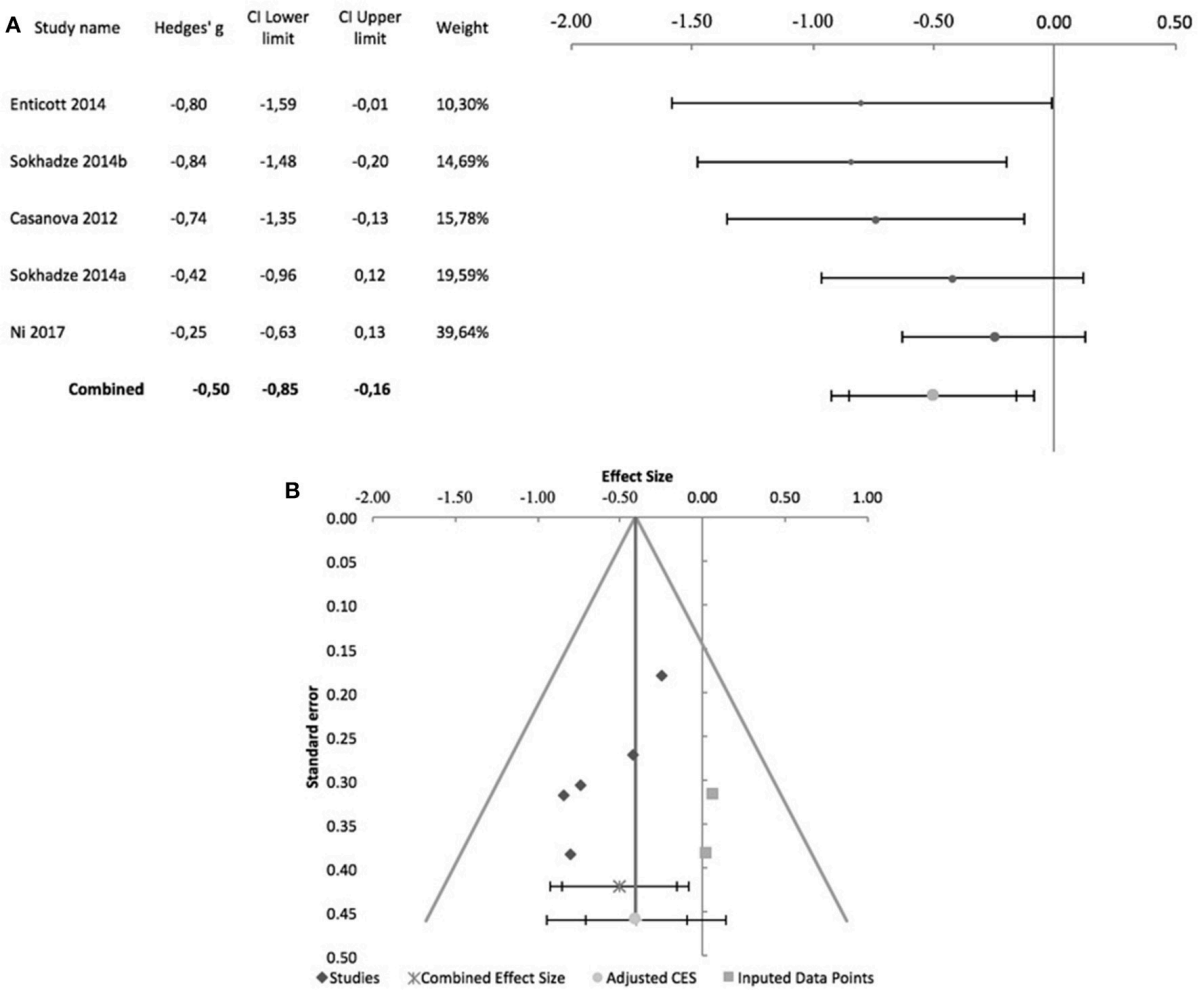
Effects of rTMS on repetitive and restricted behavior in controlled studies. **(A)** Forest-plot. Dots represent each study, with dot size reflecting study weight in the model and error bars indicating the effect size (with confidence interval). The lower line represents the combined effect size with its confidence interval (narrow interval) and its 95% prediction interval (wide interval). **(B)** Funnel-plot. There is evidence of significant publication bias. Square-shaped dots represent studies imputed by trim-and-fill analysis.

Figure 3 shows the forest plot of 5 controlled studies that assessed the effects of rTMS on stereotyped and repetitive behavior. Only one of these studies was randomized, sham-controlled and double-blind (Enticott et al., 2014). The remaining incurred in risk of bias due to lack of randomized treatment attribution or adequate blinding. Moreover, only Enticott et al. (2014) and Ni et al. (2017) adopted sham-controlled designs, the remainder comparing the active TMS arm with wait-list condition. Enticott et al. (2014) stimulated the DMPFC bilaterally at 5 Hz, using a H-coil, while Ni et al. (2017) used iTBS to stimulate both right and left DLPFC. The remaining three studies also stimulated the DLPFC bilaterally with 1 Hz rTMS. A combined effect size of −0.5 was found (95% CI: −0.85 to −0.16; *z* = −4.1; *p* < 0.001) with a non-significant Cochrane's *Q-*test (*Q* = 4.4, *p* = 0.4), *I*^2^-value of 9% and T^2^ of 0.01. The funnel plot for this combined effect size estimation was asymmetrical, and the trim-and-fill analysis was suggestive of publication bias, with two missing studies and an adjusted effect size of −0.4 (95% CI: −0.71 to −0.1; Figure 3). Egger statistic was significant (intercept = −3.5, 95%CI: −5.7 to −1.2; *t* = −4.3, *p* = 0.02).

**Figure 4:**
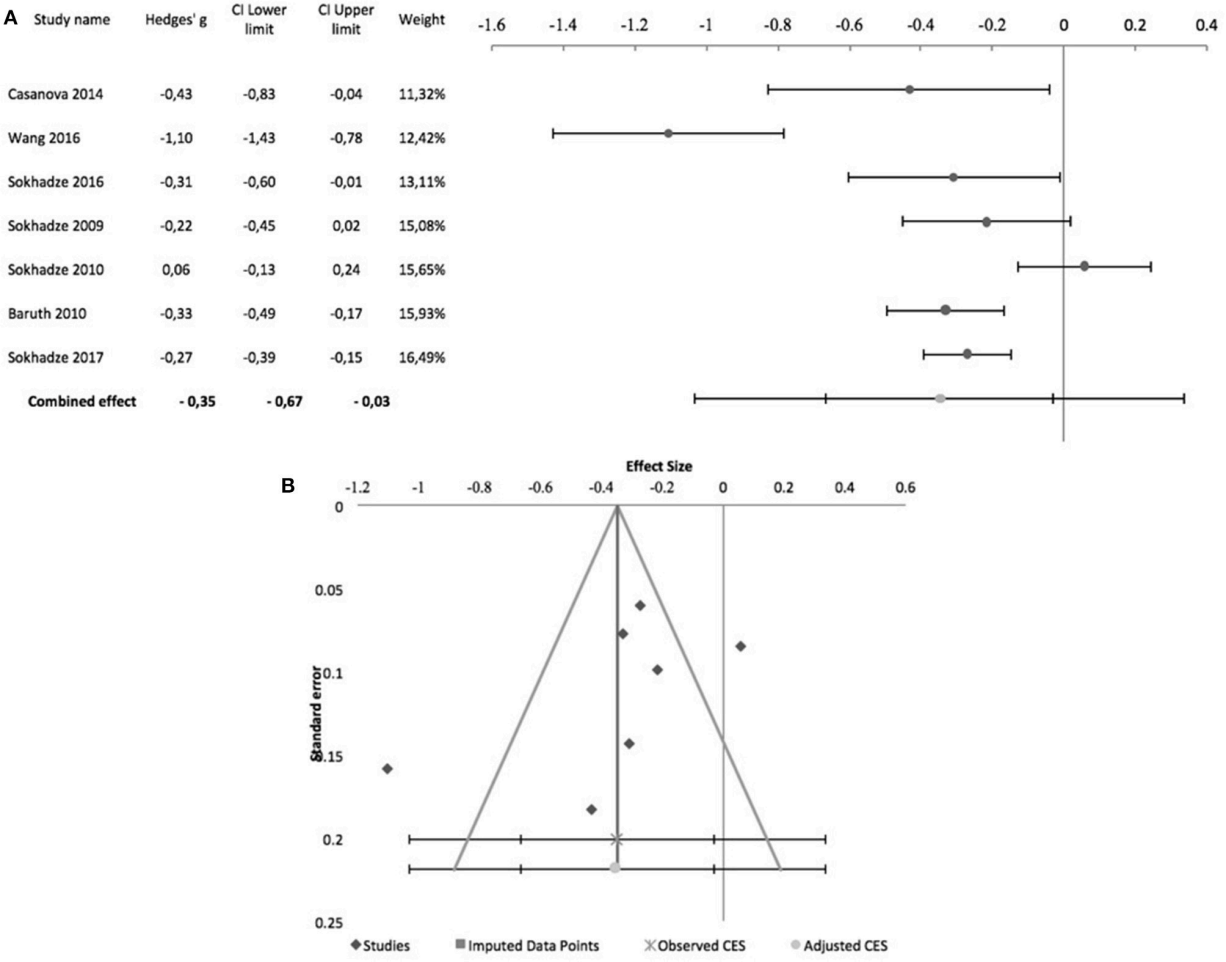
Effects of rTMS on social behavior deficits in non-controlled studies. **(A)** Forest-plot. Dots represent each study, with dot size reflecting study weight in the model and error bars indicating the effect size (with confidence interval). The lower line represents the combined effect size with its confidence interval (narrow interval) and its 95% prediction interval (wide interval). **(B)** Funnel-plot.

The effects of rTMS on the social behavior were computed based on the scores of the Social Responsiveness Scale (SRS), Lethargy and Social Withdrawal subscale of the ABC, or Social Relatedness subscale of the Ritvo Autism-Aspergers Diagnostic Scale (RAADS). Figure 4 summarizes pre-post design studies, comprising 7 studies, all by the same research group. Three of these studies included a control group but were included in this meta-analysis as no outcome data were reported for this group (Sokhadze et al., 2009, 2016; Baruth et al., 2010). None of the studies used subject or rater blinding, or an intent to treat analysis. All used stimulation frequencies between 0.5 and 1 Hz and, except for Sokhadze et al. (2009) and Sokhadze et al. (2010), all targeted the DLPFC bilaterally. These studies had a significant combined effect size of −0.35 (95% CI: −0.67 to −0.03; *z* = −2.7; *p* = 0.008). The Cochrane's *Q*-test was significant (*Q* = 44.9, *p* < 0.001), with an *I*^2^-value of 86.6% and T^2^ of 0.06. The funnel plot and trim-and-fill analysis for this combined effect size estimation were not suggestive of publication bias or missing studies (Figure 4) and the Egger statistic was not significant (intercept = −10.4, 95% CI: −26.2 to 5.4; *t* = −1.6, *p* = 0.2).

**Figure 5:**
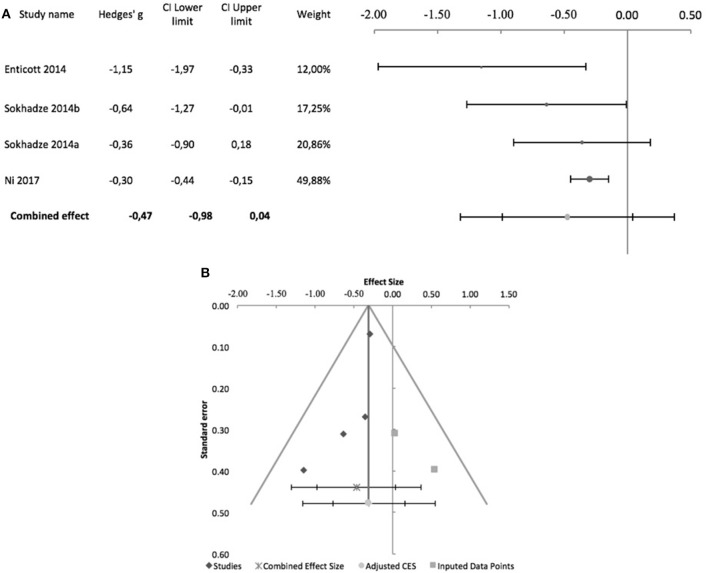
Effects of rTMS on social behavior deficits in controlled studies. **(A)** Forest-plot. Dots represent each study, with dot size reflecting study weight in the model and error bars indicating the effect size (with confidence interval). The lower line represents the combined effect size with its confidence interval (narrow interval) and its 95% prediction interval (wide interval). **(B)** Funnel-plot. There is evidence of possible significant publication bias, with 2 imputed studies (square-shaped dots) estimated by trim-and-fill analysis.

Regarding controlled studies, Casanova et al. (2012) did not report data on social behavior, and thus only four studies were entered in the meta-analysis. Only Enticott et al. (2014) and Ni et al. (2017), adopted sham-controlled designs, and only the former adopted a randomized controlled double-blind design. Regarding stimulation targets, Enticott et al. (2014) used a H-coil to stimulate the DMPFC at 5 Hz. Ni et al. (2017) used iTBS to stimulate the DLPFC, which was the target stimulated in the other two studies, albeit at low frequency (1 Hz). Figure 5 shows the forest plot of these studies, with a combined effect size of −0.47 (95% CI: −0.98 to 0.04; although *z* = −2.93; *p* = 0.003), a non-significant Cochrane's *Q*-test (*Q* = 5.4, *p* = 0.1), *I*^2^-value of 45% and T^2^ of 0.04. The funnel plot and trim-and-fill analysis were suggestive of publication bias (Figure 5), with two missing studies, but Egger statistic was not significant (intercept = −2.8, 95% CI: −6.6 to 0.9; *t* = −2.39.3, *p* = 0.1). Furthermore, one study (Enticott et al., 2014) described follow up assessments 1 month after treatment, when differences between sham and active rTMS remained significant (Hedges' g = −1.38; 95% CI: −2.27 to −0.57, *p* = 0.016).

**Figure 6:**
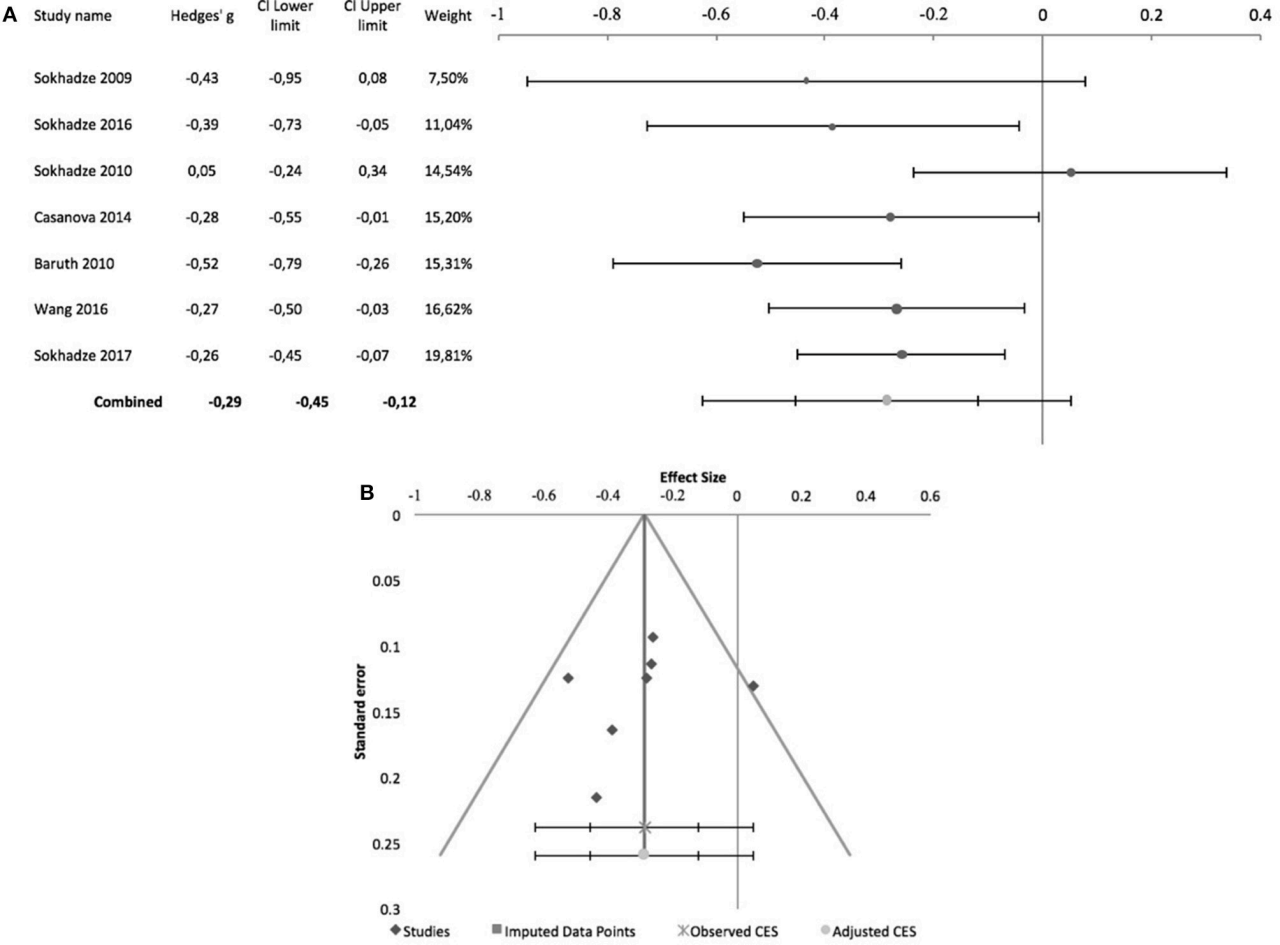
Effects of rTMS on hyperactivity in non-controlled studies. **(A)** Forest-plot. Dots represent each study, with dot size reflecting study weight in the model and error bars indicating the effect size (with confidence interval). The lower line represents the combined effect size with its confidence interval (narrow interval) and its 95% prediction interval (wide interval). **(B)** Funnel-plot.

Effects of rTMS on irritability and hyperactivity were measured with the corresponding ABC subscales. Figure 6 shows the forest plot of pre-post studies for hyperactivity, with a significant combined effect size of −0.29 (95% CI: −0.45 to −0.12; *z* = −4.2; *p* < 0.001), non-significant Cochrane's *Q*-test (*Q* = 11.3; *p* = 0.08), *I*^2^-value of 46.7% and T^2^ of 0.01. The funnel plot and trim-and-fill analysis for this combined effect size estimation were not suggestive of publication bias or missing studies (Figure 6), with a non-significant Egger statistic (intercept = −1.8, 95% CI: −9.1 to 5.5; *t* = −0.6, *p* = 0.6). All 7 of these studies were by the same research group. None of them was subject or rater-blinded, and all used low-frequency rTMS to stimulate the DLPFC, either bilaterally or unilaterally (see Table 2 for details). Only two controlled studies reported the effects of TMS on hyperactivity, both by Sokhadze and colleagues, one of which (Sokhadze et al., 2014b) found no significant differences between active TMS and waiting-list condition (Hedges' g = −0.36, 95% CI: −0.91 to 0.18) and the other (Sokhadze et al., 2014a) reporting a significant interaction between treatment arm and time (Hedges' g = −0.71, 95% CI: −1.36 to −0.09).

**Table 2:** Summary table of TMS targets and parameters.

**Source**	**Target area**	**TMS Procedure**						
		**N of sessions**	**Frequency**	**Hz**	**MT (%)**	**Pulses/session**	**Inter-train interval**	**Duration/session (minutes)**
Anninos et al., 2016	Frontal, vertex, right and left temporal, right and left parietal, occipital cortex	1 crossover session with active and sham TMS, then daily for 1 month	Daily	8–13	Not applicable	Not reported	Not reported	2
Baruth et al., 2010	Left and right DLPFC, 5 cm anterior to the site of maximal FDI stimulation	12 (6 left;6 right)	Weekly	1	90	150 (15 × 10)	20–30 s	Not reported
Casanova et al., 2012	Left and right DLPFC	12 (6 left;6 right)	Weekly	1	90	150 (15 × 10)	20–30 s	Not reported
Enticott et al., 2012	Left primary motor cortex and supplementary motor area	3	Weekly	1	100	900	Not reported	5
Enticott et al., 2014	DMPFC, coil centered and 7 cm anterior to M1, 3-4 cm from nasion	10	Daily	5	100	30 × 10	20	Not reported
Fecteau et al., 2011	Left and right pars triangularis (BA45) and pars opercularis (BA44)	4 (1 session for each target area) + 1 (fifth session of sham rTMS)	Intervals of at least 5 days, over 4 weeks	1	70	Not reported	Not reported	30
Ni et al., 2017	Left and right DLPFC; posterior superior temporal sulcus	1 session for each target area	1 week interval between sessions	50 Hz 3-pulse bursts	80 for active iTBS; 60 for sham condition (active MT)	Two courses of 600 on each hemisphere, left first, 5 minutes apart	20 trains at 10 s intervals	2 x 4
Panerai et al., 2014; Study I	Left and right premotor cortex, 2.5 cm rostral to primary motor cortex	1 session of HFrTMS LFrTMS and sham both on the left and right	Every 2 weeks	LFrTMS: 1; HFrTMS: 8	90 (resting MT)	LFrTMS: 900; HFrTMS: 30 trains of 30 stimuli each trial lasting 3.6s	56.4 s	LFrTMS: 15; HFrTMS: 30
Panerai et al., 2014; Study II	Left premotor cortex, 2.5 cm rostral to primary motor cortex	10 (HFrTMS, LFrTMS)	Every weekday (10 days), over 2 weeks	LFrTMS: 1; HFrTMS: 8	90 (resting MT)	LFrTMS: 900; HFrTMS: 30 trains of 30 stimuli each trial lasting 3.6s	56.4 s	LFrTMS: 15; HFrTMS: 30
Panerai et al., 2014; Study III	Left premotor cortex, 2.5 cm rostral to primary motor cortex	4 HFrTMS or sham; Crossover design TMS-sham-TMS-sham	Daily (5 days)	LFrTMS: 1; HFrTMS: 8	90 (resting MT)	LFrTMS: 900; HFrTMS: 30 trains of 30 stimuli each trial lasting 3.6s	56.4 s	LFrTMS: 15; HFrTMS: 30
Panerai et al., 2014; Study IV	Left premotor cortex, 2.5 cm rostral to primary motor cortex	10 HFrTMS or 10 HFrTMS+ EHI	Every weekday (10 days), over 2 weeks	8	90 (resting MT)	LFrTMS: 900; HFrTMS: 30 trains of 30 stimuli each trial lasting 3.6s	56.4 s	LFrTMS: 15; HFrTMS: 30
Sokhadze et al., 2009	Left DLPFC, 5 cm anterior to maximal FDI response	6	Twice a week	0.5	90	150 (15 × 10)	20-30s	Not reported
Sokhadze et al., 2012	Left and right DLPFC	12 (6 left;6 right)	Weekly	1	90	150 (15 × 10)	Not reported	Not reported
Sokhadze et al., 2014b	Right and left DLPFC, 5 cm anterior to maximal FDI response	18 (6 left, 6 right, 6 bilaterally) + NFB (20 minutes)	Weekly	1	90	180	Not reported	Not reported
Sokhadze et al., 2014a	Left and right DLPFC	18 (6 right, 6 left, 6 alternating right and left)	Weekly	1	90	180 (9 × 20)	Not reported	Not reported
Sokhadze et al., 2016	Right and left DLPFC, 5 cm anterior to maximal FDI response	18 (6 right, 6 left, 6 bilaterally)	Weekly	1	90	180 (9 × 20)	20–30 s	Not reported
Abujadi et al., 2017	Right DLPFC	15	5 days a week, over 3 weeks	50 Hz 3-pulse bursts at 5 Hz	100	900 (300 bursts)	8 s On/2 s Off	5
Casanova et al., 2014	Left and right DLPFC	18 (6 left, 6 right, 6 bilaterally)	Weekly	0.5	90	160 (8 × 20)	Not reported	Not reported
Gómez et al., 2017	Left DLPFC	20	Daily	1	90 (resting MT)	1500 (4 × 375)	60 s	30
Sokhadze et al., 2010	Left DLPFC, 5 cm anterior to maximal FDI response	6	Twice a week	0.5	90	150 (15 × 10)	Not reported	Not reported
Sokhadze et al., 2017	Bilateral DLPFC, 5 cm anterior to and in a parasagittal plane to the site of maximal abductor pollicis brevis stimulation	18	Weekly	0.5	90 (resting MT)	160 (8 × 20)	20 s	Not reported
Wang et al., 2016	Left and right DLPFC, 5 cm anterior to the site of maximal FDI stimulation	12 (6 left, 6 right)	Weekly	0.5	90	160	20–30 s	Not reported
Avirame et al., 2017 case 1	Bilateral medial prefrontal cortex	27	Daily, over 6 weeks (missed 3 treatments)	5	Not reported	60 × 10	20 s	30
Avirame et al., 2017 case 2	Bilateral medial prefrontal cortex	29	Daily, over 6 weeks (missed 1 treatment)	5	Not reported	60 × 10	20 s	30
Cristancho et al., 2014	Left and right DLPFC	36 (10 right, 26 left)	Right: daily; Left: after 20 sessions, TMS was tapered over 3 weeks	1	90 (resting MT)	Right: 150 PPS increased to 300 PPS in the second week; Left: 300 PPS increased to 600 PPS in the fourth week	Right: 10s On/10−30s Off; Left: 10s On/ 10−15s Off	Not reported
Enticott et al., 2011	Bilateral medial prefrontal cortex	9	Every weekday (10 days), over 2 weeks	5	100	30 × 10	20s	15
Niederhofer, 2012	Supplementary motor area	5	Daily	1	Not reported	1,200	Not reported	60

*DLPFC, Dorsolateral prefrontal cortex; DMPFC, Dorsomedial prefrontal cortex; FDI, First Dorsal Interosseous muscle; HFrTMS, High-frequency repetitive transcranial magnetic stimulation; iTBS, Intermittent Theta-burst stimulation; LFrTMS, Low-frequency repetitive transcranial magnetic stimulation; MT, Motor threshold; NFB, Neurofeedback; PPS, Pulses Per Session; rTMS, Repetitive transcranial magnetic stimulation; TMS, Transcranial magnetic stimulation*.

**Figure 7:**
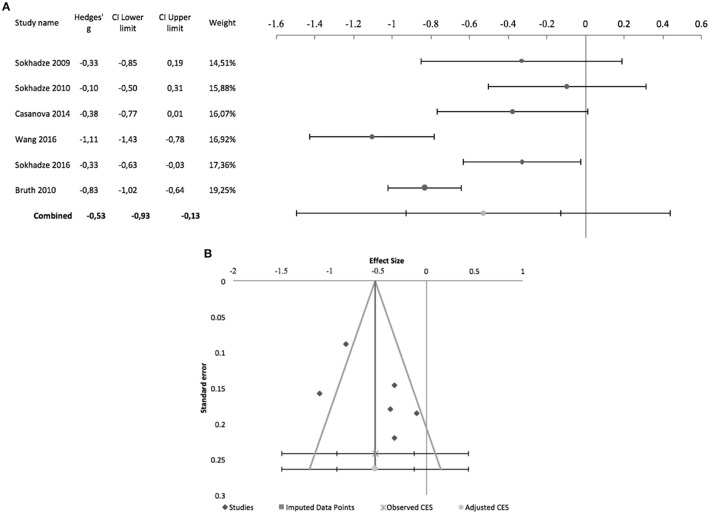
Effects of rTMS on irritability in non-controlled studies. **(A)** Forest-plot. Dots represent each study, with dot size reflecting study weight in the model and error bars indicating the effect size (with confidence interval). The lower line represents the combined effect size with its confidence interval (narrow interval) and its 95% prediction interval (wide interval). **(B)** Funnel-plot.

**Figure 8:**
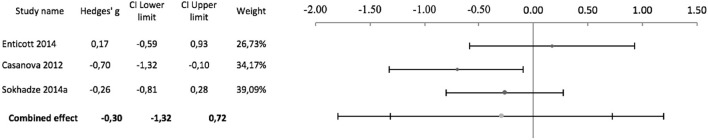
Forest-plot of controlled studies that assessed the effects of rTMS on irritability. Dots represent each study, with dot size reflecting study weight in the model and error bars indicating the effect size (with confidence interval). The lower line represents the combined effect size with its confidence interval (narrow interval) and its 95% prediction interval (wide interval).

Figure 7 shows the forest plot of pre-post studies regarding irritability, excluding Sokhadze et al. (2017) who did not provide data on this measure. Again, these 6 studies were all by the same research group and all incurred in risk of bias due to lack of blinding (see above and Table 2 for further details) A significant combined effect size of −0.53 was found (95% CI: −0.93 to −0.13; *z* = −3.4; *p* = 0.001) with a significant Cochrane's *Q*-test (*Q* = 30.8; *p* < 0.001), *I*^2^-value of 83.8% and T^2^ of 0.1. The funnel plot and trim-and-fill analysis for this combined effect size estimation were not suggestive of publication bias or missing studies (Figure 7) and the Egger statistic was not significant (intercept = 11.5, 95% CI: −11 to 33.9; *t* = 1.3, *p* = 0.3). Figure 8 shows the forest plot of controlled studies that assessed the effects of TMS on Irritability, excluding Ni et al. (2017) and Sokhadze et al. (2014b) who did not provide data on this outcome. From these 3 studies, only Enticott et al. (2014) had a randomized, sham-controlled, double-blind design, while the remaining two compared active rTMS with wait-list condition (see above for stimulation parameter details). Their combined effect size was −0.3 (95% CI: −1.32 to 0.72; *z* = −1.3; *p* = 0.2), with a non-significant Cochrane's *Q*-test (*Q* = 3.3, *p* = 0.2), *I*^2^-value of 39.9% and T^2^ of 0.06. Funnel plot and Egger statistic were not interpretable due to the low number of studies (data not shown).

#### Effects on cognitive function

Eleven studies assessed the effects of rTMS on cognitive performance in ASD patients, eight of which were controlled studies comparing active rTMS treatment with either waiting-list controls (Sokhadze et al., 2009, 2012, 2014a,b; Baruth et al., 2010; Casanova et al., 2012) or sham-rTMS (Fecteau et al., 2011; Ni et al., 2017). The 3 remaining studies, comparing cognitive performance before and after rTMS, could not be used for meta-analysis because a common cognitive domain was not assessed in all of them (Sokhadze et al., 2010, 2016; Abujadi et al., 2017). Most of these studies report performance measures on a visual oddball-type task, which requires subjects to respond (e.g., press a button), or omit a response, when an infrequent stimulus appears within a series of frequent standard stimuli, presented rapidly. This type of task loads on several dimensions of executive control, namely attention control, working memory, response inhibition, and ability to shift between alternative response strategies (Huettel and McCarthy, 2004). Performance is conventionally described in terms of reaction-time do target stimuli, omission errors (failing to respond to the target), commission errors (responding to non-targets), and total number of errors. Ni et al. (2017) and Abujadi et al. (2017) used the Wisconsin Card Sorting Test, a classical and widely known neuropsychological test used to assess cognitive set-shifting ability. Abujadi et al. (2017) additionally used the Stroop color-word test, a classical interference control task, while Fecteau et al. (2011) used the Boston Naming Test, a widely used test of confrontational naming, to assess naming skills.

**Figure 9:**
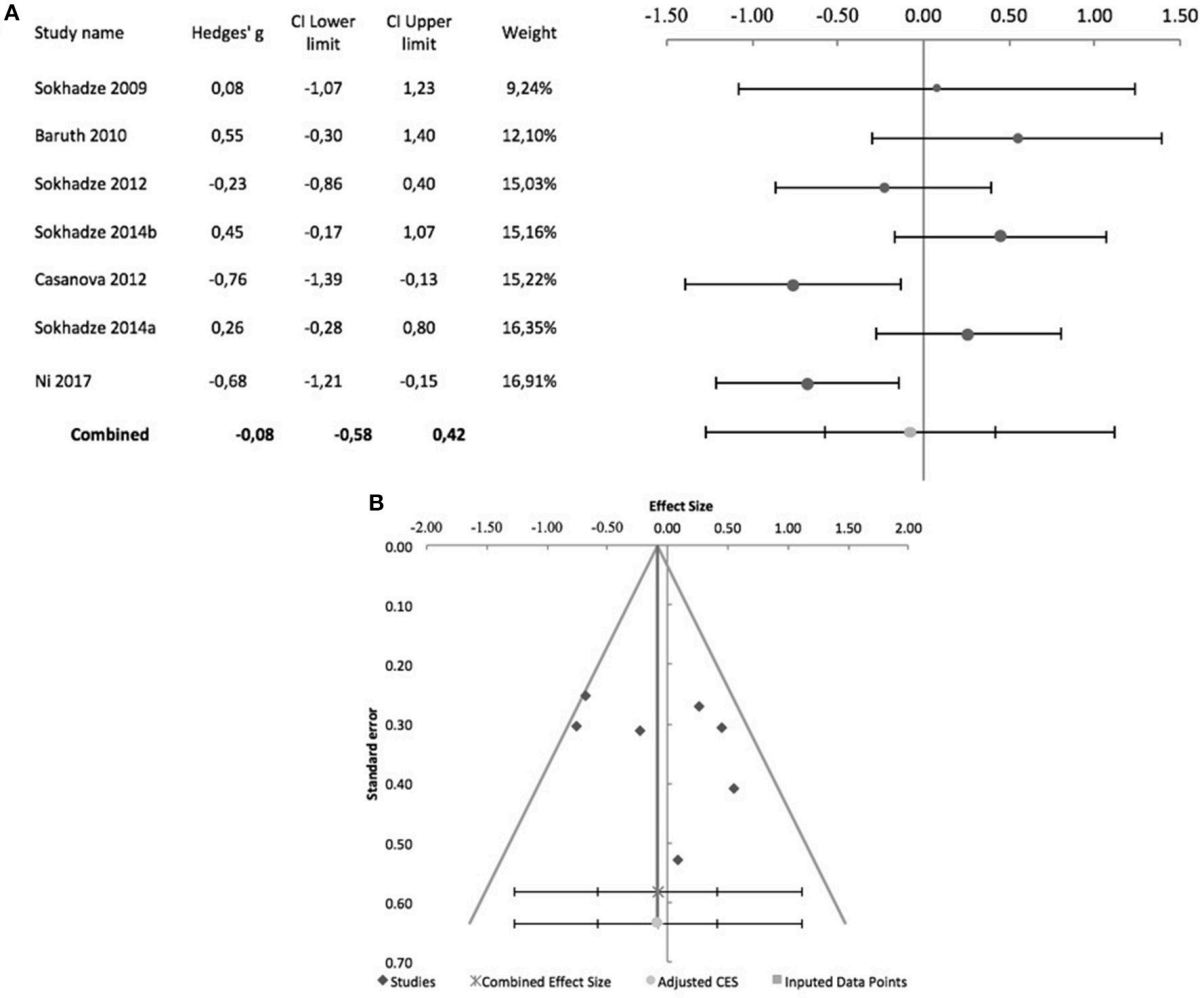
Effects of rTMS on reaction time during performance of executive control tasks, in controlled studies. **(A)** Forest-plot. Dots represent each study, with dot size reflecting study weight in the model and error bars indicating the effect size (with confidence interval). The lower line represents the combined effect size with its confidence interval (narrow interval) and its 95% prediction interval (wide interval). **(B)** Funnel-plot.

**Figure 10:**
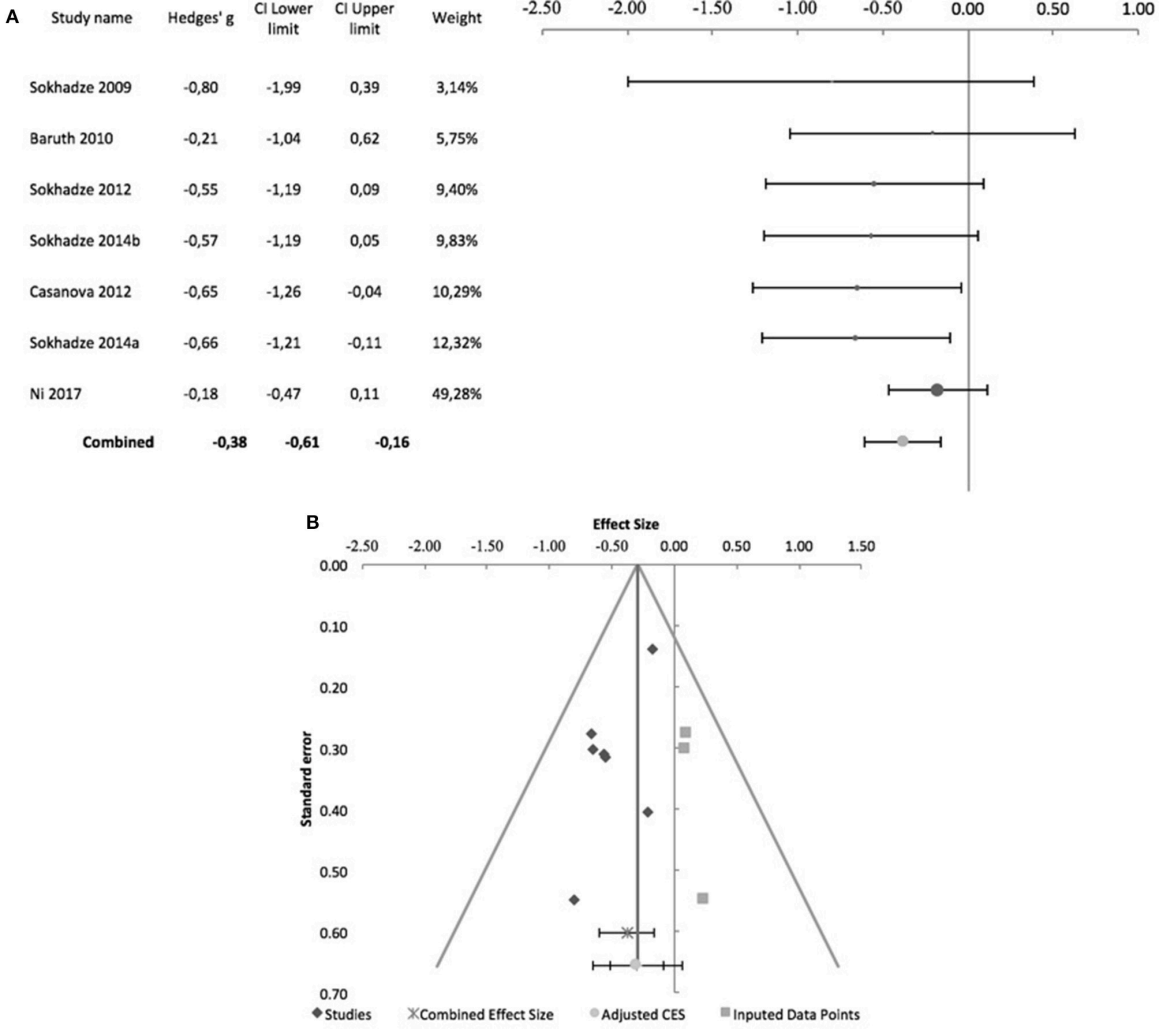
Effects of rTMS on total number of errors during performance of executive control tasks. **(A)** Forest-plot. Dots represent each study, with dot size reflecting study weight in the model and error bars indicating the effect size (with confidence interval). The lower line represents the combined effect size with its confidence interval (narrow interval) and its 95% prediction interval (wide interval). **(B)** Funnel-plot. There is evidence of possible significant publication bias, with 3 imputed studies (square-shaped dots) estimated by trim-and-fill analysis.

Sokhadze et al. (2010, 2016) while reporting no change in reaction time in oddball-type tasks after rTMS in their ASD samples, did not provide details on the underlying data, and meta-analysis was not conducted. Figure 9 shows the forest plot of the 7 controlled studies assessing the effects of rTMS on reaction time to target stimuli. All were by the same research group except Ni et al. (2017), which was also the only that used iTBS and a sham-treated control group. The remaining studies all used low-frequency rTMS. All studies stimulated the DLPFC bilaterally, with the exception of Sokhadze et al. (2009) who only stimulated the left DLPFC. None of the seven studies used subject or rater-blinding, and thus all were exposed to both performance and detection bias. The combined effect size of −0.08 was not significant (95% CI: −0.58 to 0.42; *z* = −0.4; *p* = 0.7), with a significant Cochrane's *Q*-test (*Q* = 17.66, *p* = 0.007), *I*^2^-value of 66% and T^2^ of 0.2. The funnel plot and trim-and-fill analysis were not suggestive of publication bias and suggested no missing studies (Figure 9), and the Egger statistic was not significant (intercept = 3.42; 95% CI: −6.74 to 13.31; *t* = 0.9, *p* = 0.4). Figure 10 shows the forest plot of the same controlled studies regarding the effects of rTMS on the number of errors in either a visual oddball task or the Conner's Continuous Performance Test, excluding Fecteau et al. (2011) that did not assess this cognitive domain. The combined effect size of−0.38 was significant (95% CI: −0.61 to −0.16; *z* = −4.2; *p* < 0.001), with a non-significant Cochrane's Q test (*Q* = 5.6, *p* = 0.5), *I*^2^-value of 0% and T^2^ of 0. The funnel plot and trim-and-fill analysis were suggestive of publication bias, with three missing studies to the right of the adjusted combined effect size (Figure 10), and an adjusted effect size of −0.3 (95% CI: −0.5 to −0.08). Egger statistic was significant (intercept = −0.1.61; 95% CI: −3.1 to −0.1; *t* = −2.6, *p* = 0.048). Non-controlled studies reported consistent results, with Sokhadze et al. (2010) describing a significant reduction in the total percentage of errors from 11 ± 12.3 to 3.3 ± 3.2% (*t* = 2.4, *p* = 0.04), and the same group showing a similar result (mean change in % total errors of−5.5 ± 14.04, *t* = −2.1, *p* = 0.047) in a later study (Sokhadze et al., 2016).

#### Other effects

In a randomized, sham-controlled study, Enticott et al. (2014) assessed the effects of deep rTMS on performance of a group of tasks loading on theory of mind skills (Reading the Mind in the Eyes Task and Animations Mentalizing Task). After 10 sessions of 5 Hz rTMS, delivered bilaterally over the dorsomedial prefrontal cortex at 100% of the motor threshold, no differences in performance were found with regards to the sham control in any of the tasks (respectively Hedges g = 0.5, 95% CI: −0.28 to 1.32; and Hedges' g = 0.71, 95% CI: −0.08 to 1.54). In an earlier study, the same group (Enticott et al., 2014) assessed the effects of a single 1 Hz rTMS session, delivered either to the left primary motor cortex or the left supplementary motor area, when compared to sham stimulation of the left primary motor cortex. Clinical outcome measures were motor reaction time to a stimulus and movement time, none of which showed any significant changes after rTMS to either target.

Panerai et al. (2014) performed a series of small proof of principle trials to assess the utility of rTMS for improving eye-hand integration ability in adolescents with ASD and severe cognitive delay. In the first of these trials the authors tested the effects of several stimulation targets and parameters (1 Hz rTMS, 8 Hz rTMS and sham), one session of each over the left and right premotor cortex, at 2-week intervals. The authors found a significant effect of 8 Hz rTMS over the left premotor cortex, when compared both to sham and 1 Hz rTMS. In the second trial, each of the treatment conditions (1 Hz rTMS, 8 Hz rTMS or sham) was applied over the left premotor cortex to three different groups, for a total of 10 sessions over consecutive weekdays. A significantly better performance was reported in the group treated with 8 Hz rTMS when compared to the other two groups. The third trial used a complex design to assess long-lasting effects of 8 Hz rTMS: the authors found a significant effect when compared to sham stimulation, but the effect only persisted for 1 h after treatment conclusion. In a final trial the authors report long-lasting effects of 8 Hz rTMS at 4 weeks follow-up, but only when rTMS was combined with an eye-hand integration training program, and not for rTMS alone or the training program alone.

#### Case-reports

Four case-reports, describing five patients successfully treated with rTMS (three of them with deep rTMS) are summarized in Tables 1–3.

**Table 3:** Summary of study outcome measures and results, including follow-up and adverse effects.

**Source**	**Behavioral measures**	**Cognitive measures**	**Behavioral Results**	**Cognitive Results**	**Adverse reactions**	**Follow up**	**Additional comments**
Anninos et al., 2016	Deficits in social communication and interaction, imaginative play, or making friends; intellectual disability		Active treatment: 4 patients showed major changes; 3 minor changes and 1 mixed changes in the list of disorders. No changes in the sham group		Not reported	No follow up after stimulation	
Baruth et al., 2010	ABC (Irritability and Hyperactivity Subscales); SRS (Social Awareness Subscale); RBS-R	Kanizsa Illusory Figure Test: RT, error rates (commision, omission, total), post-error RT	1-ABC: significant reduction in repetitive behavior and irritability subscales. No changes in hyperactivity or social awareness subscales. 2- RBS-R: reduction in repetitive and restricted behavior patterns.3- SRS not reported	No significant differences in RT and Errors, in any of the groups	5/16 itching sensation around nose during stimulation; 1/16 reported mild, transient tension-type headache after stimulation	No follow up after stimulation	
Casanova et al., 2012	ABC; SRS (Social Awareness Subscale); RBS-R stereotyped (self-injurious, compulsive, ritualistic, sameness, and restricted range subscales)	Kanizsa Illusory Figure Test: RT, error rate (commission, omission, total error rate)	1-ABC: significant reduction in irritability subscale post- TMS. No changes in hyperactivity subscale. 2-RBS-R: significant decrease in repetitive and restricted behavior patterns post-TMS. 3-SRS: No changes in social awareness after TMS. Waiting-list: no significant changes in any measure	1-RT: no significant change as a result of rTMS. 2-Omission error rate: significant decrease in TMS group in comparison to waiting list. 3-Total error rate: significant decrease post TMS	Not reported	No follow up after stimulation	
Enticott et al., 2012		RT; Movement time		1-RT: no significant difference post-TMS. 2- Movement time: no significant difference post-TMS but significant reduction after sham stimulation	Not reported	No follow up after stimulation	
Enticott et al., 2014	RAADS;ASQ; IRI	Reading the mind in the eyes test; animations mentalizing test	1-RAADS: significant reduction on the social relatedness subscale post-TMS, but not post-sham. Significant time condition interaction for the active condition. 2- IRI: significant reduction on the fantasy subscale post-TMS, but not post-sham. 3- ASQ: No effect	No significant effects of condition (TMS or sham) in mentalizing measures	1/15 lightheadedness for 5 minutes after TMS; 2/15 minor facial discomfort during TMS	1 month follow-up: significant reduction in social relating symptoms in TMS participants relative to sham participants	40% of TMS group and 38% of sham group on psychotropic medication (mostly antidepressants)
Fecteau et al., 2011		Boston Naming Test		Worse performance following TMS to the left pars opercularis; better performance after TMS to the left pars triangularis than after sham stimulation	2/10 sleepy; 1/10 more emotional; 1/10 stiff neck; 1/10 dizziness	No follow up after stimulation	Neuronavigation guided
Ni et al., 2017	Y-BOCS; SRS	CCPT; WCST	1-Y-BOCS: compulsive behaviors significantly decreased at 8 h and 2 days after posterior superior temporal sulcus iTBS compared to sham (parent-reports). No change in self-reported scores. No difference between DLPFC and sham stimulation. 2-SRS: improvement in parent-reported scores of social communication deficits 8 days after DLPFC iTBS compared to sham. No change in self-reported scores	1-CCPT: decrease in RT, omission errors, and commission errors post- iTBS over the DLPFC. Significant reduction in RT after DLPFC iTBS compared to sham, but no differences in errors. 2-WCST: no significant changes after iTBS	3 participants felt transient discomfort during iTBS over the DLPFC because of muscle twitches around the eyes	2 days follow-up: significant reduction in parent-reported Y-BOCS compulsion subscale scores	1 patient on sertraline; 1 patient on fluoxetine; 1 patient on methylphenidate
Panerai et al., 2014; Study I		Number of successes in eye–hand integration tasks from PER-P		HFrTMS: increase in eye–hand integration after TMS to left premotor cortex. LFrTMS and sham: no differences in eye–hand integration. LFrTMS, HFrTMS, and sham pairwise comparisons: significant difference between HFrTMS and both LFrTMS and sham	Not reported	No follow up after stimulation	
Panerai et al., 2014; Study II		Number of successes in eye–hand integration tasks from PER-P		LFrTMS, HFrTMS, and sham: highest increase in mean performance with HFrTMS, followed by LFrTMS and sham. Pre-post comparisons showed difference only for HFrTMS	Not reported	No follow up after stimulation	
Panerai et al., 2014; Study III		Number of successes in eye–hand integration tasks from PER-P		Significant increase in eye-hand integration after TMS, in comparison to sham	1/6 children developed increased restlessness and rapid mood swings during the first experimental condition	2,5 days follow up; HfrTMS showed no difference from sham TMS or from baseline assessment	
Panerai et al., 2014; Study IV		Number of successes in eye–hand integration tasks from PER-P		Pairwise comparisons showed a statistical difference between HFrTMS+Eye–hand integration training and both treatments alone. No difference between Eye–hand integration training and HFrTMS	Not reported	1 month follow up; TMS+training significantly better than either intervention alone after 1 week; at 2 weeks TMS+training superior to training alone; at 4 weeks no differences between groups	
Sokhadze et al., 2009	ABC; SRS; RBS-R; CGI	Kanizsa Illusory Figure Test: RT, error rate (commission, omission, total errors)	1- RBS-R: only TMS group reported. Significant reduction in repetitive behavior subscale. No significant differences in other RBS-R subscales. 2- ABC/SRS/CGI: not reported	No significant differences in RT and Errors, in any of the groups	None	No follow up after stimulation	
Sokhadze et al., 2012		3-category odd-ball task, RT, error rate (commission, omission and total errors), post-error RT		1- RT: no change; 2- Omission error rate: significant decrease post-TMS, but not post waiting period. 3- Commission error rate: No between group differences. 4- Post-error RT: slowing of post-error RT in TMS group compared to waiting list group	Not reported	No follow up after stimulation	
Sokhadze et al., 2014b	ABC (Irritability, Lethargy/Social Withdrawal and Hyperactivity subscales); RBS-R	Three-Stimuli Oddball Task: RT, error rates (commission, omission, total), post-error RT	1- ABC: significant reduction in Lethargy/Social Withdrawal and hyperactivity subscales. 2- RBS-R: significant decrease in total RBS-R score and in stereotypic behavior and ritualistic/sameness behavior subscales. No significant changes in waiting list group in any measure	1- RT: no change. 2- Omission error rate: no differences between groups. 3- Commission error rate: significant decrease post TMS-NFB. 4-Total error rate: significant decrease post TMS-NFB. 5- Post-error RT: TMS-NFB group presented a slowing of post-error RT; waiting list group showed no changes	None	No follow up after stimulation	
Sokhadze et al., 2014a	ABC; SRS; RBS-R	Visual odd-ball task: RT, error rate (commission, omission, total error rate), post-error RT	1- ABC: significant reduction in irritability, lethargy/social withdrawal and hyperactivity. 2- RBS-R: significant decrease in total RBS-R score, stereotypic behavior subscale and ritualistic/sameness behavior. Waiting list: no change in any of the scales	1- RT: no significant changes post TMS. 2: Omission error rate: No between group differences. 3-Comission error rate: significant decrease post-TMS, WTL not reported. 4- Post-error RT: TMS group showed post-error RT increase	Not reported	No follow up after stimulation	
Sokhadze et al., 2016	ABC; RBS-R	Three-stimuli oddball task: RT, error rates, post-error RT	1- ABC: significant reduction in irritability, lethargy/social withdrawal and hyperactivity subscales. 2- RBS-R: significant decrease in total RBS-R score, stereotypic behavior, ritualistic/sameness behavior and compulsive behavior subscales	1- RT: no significant change. 2: Total error rate: significant decrease mainly due to decrease in commission errors. 3- Post-error RT: pretreatment speeding changed into post-error slowing	Not reported	Every participant was evaluated before TMS course and within 2 weeks following TMS treatment	
Abujadi et al., 2017	RBS-R; Y-BOCS	WCST; Stroop test	1- RBS-R: significant decrease in mean scores post treatment. 2- Y-BOCS: decrease in mean overall compulsive behaviors post treatment	1-WSCT: improvement in perseverative erros post treatment. 2- Stroop test: improvement in total time for completion post treatment	None	5 patients were assessed at 5 months follow up: improvement in all the scales was maintained, except for the stroop test	Neuronavigation guided;
Casanova et al., 2014	ABC; SRS; RBS-R		1- ABC: significant reduction in irritability, lethargy/social withdrawal and in hyperactivity subscales. 2- RBS-R: significant decrease in total RBS-R score, stereotypic behavior subscale and ritualistic/sameness behavior. 3-SRS not reported		Not reported	Every participant was evaluated before TMS course and within 2 weeks following TMS treatment	
Gómez et al., 2017	ABC; ADI-R; ATEC; GCIS		ABC/ADI-R/ATEC/GCIS: significant decrease in the total scores one month after the intervention		Not reported	Follow up to 6 months: decrease in ABC, ADI-R and ATEC remained significant	
Sokhadze et al., 2010	ABC; SRS; RBS-R	Three-Stimuli Visual Oddball with Novel Distracters: RT, error %	1- RBS-R: significant reduction in repetitive behavior subscale. No significant differences in other RBS-R subscales. 2- ABC/SRS: not reported	1- Error %: significant reduction post TMS. 2-RT: no difference	None	Participants were evaluated prior to receiving TMS and 2 weeks following treatment	
Sokhadze et al., 2017	ABC; RBS-R; SRS		1- ABC: significant reduction in lethargy, hyperactivity and speech subscales. No changes in stereotyped behavior. 2- RBS-R: significant decrease in RBS-R total score, stereotypic, ritualistic and compulsive behavior. 3-SRS: significant improvement in social awareness, social cognition and social motivation		Not reported	No follow up after stimulation	
Wang et al., 2016	ABC; RBS-R		1- ABC: significant reduction in stereotyped behavior, hyperactivity and inappropriate speech. 2- RBS-R: significant decrease in total RBS-R, and ritualistic/sameness, stereotypical and compulsive behavior subscales		None	No follow up after stimulation	
Avirame et al., 2017, case 1	IRI; Autism Spectrum in vocal aspects	Computarized Cognitive Battery (Mindstream, Neurotrax) and a battery of emotional recognition tasks from the Autism research center in Cambridge (eye test, face tests, CAM face-voice)	1-IRI/Autism Spectrum in vocal aspects: improvement in autistic symptoms and empathy. 2-Y-BOCS: decrease in OCD symptoms. 3-Eyes test/CAM voice: reduction in error rate. 4-Faces test and CAM face: no effect	1-Mindstream: increase in global scores, executive function, attention, speed processing, motor skills, memory and verbal fluency scores	Not reported	2 month follow up (self-assessment questionnaires): Obsessive compulsive disorder symptoms were still significantly lower than baseline, in both cases	
Avirame et al., 2017, case 2			1-IRI/Autism Spectrum in vocal aspects: improvement in autistic symptoms and empathy. 2-Y-BOCS: decrease in OCD symptoms. 3-Eyes test/CAM voice/CAM face: reduction in error rate. 4-Faces test: no difference in error rate	1-Mindstream: increase in global scores, executive function, attention, speed processing, visual spacial and motor skills scores; decrease in memory and verbal fluency scores	Not reported		
Cristancho et al., 2014			Improvement in patient's behavior, social interactions, ability to cope with change and ability to concentrate in school		Mild headaches, jaw twitch during stimulation and transient dizziness immediately after treatments	No follow up after stimulation	Patient on olanzapine/ fluoxetine, guanfacine, clonazepam
Enticott et al., 2011	IRI; ASQ; RAADS		Decrease in all the scales after treatment		None	1 month follow up: sustained improvement in ASQ and RAADS scores	
Niederhofer, 2012	ABC		Improvement in ABC irritability and stereotypy scales, whereas social withdrawal and inappropriate speech-associated items did not show any benefit		Not reported	No follow up after stimulation	

*ABC, Aberrant Behavior Checklist; ADI-R, Autism Diagnostic Interview - Revised; ASQ, Autism Spectrum Quotient; ATEC, Autism Treatment Evaluation Checklist; CAM, Cambridge mindreading face-voice tests; CCPT, Conners' Continuous Performance Test; CGI, Clinical Global Impression Scale; DLPFC, Dorsolateral prefrontal cortex; GCIS, Global Clinical Impression Scale; HFrTMS, High-frequency repetitive transcranial magnetic stimulation; IRI, Interpersonal Reactivity Index; iTBS, Intermittent Theta-burst stimulation; LFrTMS, Low-frequency repetitive transcranial magnetic stimulation; NFB, Neurofeedback; RAADS, Ritvo Autism-Aspergers Diagnostic Scale; RBS-R, Repetitive Behavior Scale; RT, Reaction time; rTMS, Repetitive transcranial magnetic stimulation; PEP-R, Psychoeducational Profile-Revised; TMS, Transcranial magnetic stimulation; SRS, Social Responsiveness Scale; WCST, Wisconsin Card Sorting Test; Y-BOCS, Yale-Brown Obsessive Compulsive Scale*.

#### Adverse effects

No serious adverse effects were reported. On the whole, 19 patients reported transient discomfort, during or immediately after rTMS. Facial discomfort during treatment was by far the most common side-effect, especially in patients treated with TBS (11 patients). While, overall, adverse effects seem to be infrequent, a more accurate estimation of their true incidence in treated patients was not possible due to the fact that many reports omit any mention to adverse effects, and it is thus unclear whether they did not occur or were simply under-reported.

## Discussion

### Summary of main findings

Here we conducted a systematic review of the available literature on the use of TMS to treat ASD. We found 23 studies describing the effects of rTMS on ASD symptoms and ASD-related cognitive deficits, 4 of which were case-reports, 7 were non-controlled prospective intervention trials and the 12 were controlled clinical trials. Most studies assessed the typical autistic syndrome dyad of social interaction deficits and repetitive and stereotyped behavior. In terms of cognitive function, most studies assessed executive functioning using paradigms that load on response inhibition, working memory, and flexibility.

Meta-analyses of these studies, conducted separately for controlled and non-controlled studies, globally showed that rTMS treatment results in a significant reduction of repetitive behavior, with a medium-size effect size, but with evidence of significant publication bias in both meta-analyses, tempering the potential enthusiasm with this finding. The effects of rTMS on social interaction deficits were less robust, with non-controlled studies showing a small to medium effect size, albeit with significant heterogeneity, while controlled studies, although with a significant z-statistic suggesting a significant combined effect size, had a 95% CI, estimated using the weighted variance method, crossing the line of no-effect. This results from the fact that the conventional z-distribution method underestimates confidence interval width when the number of studies is small and heterogeneity moderate or high, as in the case here (Sánchez-Meca and Marín-Martínez, 2008). Moreover, there was evidence for publication bias in the controlled studies meta-analysis. Altogether, evidence for a significant effect of rTMS treatment on social interaction deficits is thus modest and inconsistent, with a clear need for more robust evidence before a definitive conclusion is reached. Similarly, evidence of significant effects on hyperactivity or irritability is limited, with either small effect-sizes, or significant heterogeneity that limits the interpretability of combined effect-sizes.

With respect to the effects of rTMS on cognitive function, available evidence suggests a positive effect on executive and attentional control, with a reduction of the total number of errors in specific oddball-type cognitive tests. The combined effect size of these studies was of moderate magnitude, with no evidence of heterogeneity, but significant evidence of publication bias. In contrast, rTMS appears to have no significant effect on reaction time, a correlate of the psychomotor slowness that is characteristic of ASD. One sham-controlled study explicitly studied the effects of deep rTMS on the ability to solve Theory of Mind problems and found no significant improvement in this complex cognitive skill after TMS (Enticott et al., 2011). Another study assessed the effects of rTMS on eye-hand integration ability and found a significant improvement in performance of eye-hand integration tasks (Panerai et al., 2014). Finally, Fecteau et al. (2011) found that low-frequency rTMS over the left pars triangularis resulted in better performance of a confrontational naming task than sham-stimulation or rTMS over the homolateral pars opercularis. These are isolated proof-of-concept studies that measured the effects of rTMS on specific cognitive functions relevant in the context of ASD, but that did not see any further developments to date.

The choice of stimulation parameters and targets for rTMS in ASD treatment seems to follow a pattern whereby low-frequency stimulation, with suppressive effects on cortical excitability, is used for dorsolateral prefrontal targets while medial prefrontal targets and other, non-prefrontal targets, are preferentially stimulated with higher frequencies (5–10 Hz), which facilitate cortical excitability. In addition to safety concerns regarding the use of TMS on a vulnerable population such as ASD patients, the rationale invoked in studies using low-frequency rTMS to target the DLPFC rests on the hypothesis of an elevated local cortical excitation-to-inhibition ratio in ASD, and the underlying theory of decreased GABAergic dampening of cortical excitability (Casanova et al., 2002; Oblak et al., 2010). However, both Abujadi et al. (2017) and Ni et al. (2017) used iTBS (with predominantly facilitatory effects) over the right DLPFC and obtained significant improvements in executive cognitive function and, in one of the studies (Abujadi et al., 2017), also in stereotyped and repetitive behavior. In fact, there are arguments to support that iTBS potentiates both cortical excitation and inhibition, the latter through mechanisms that may differ from those involved in low-frequency rTMS (Rossi et al., 2009; Abujadi et al., 2017; Ni et al., 2017). On the other hand, studies targeting the DMPFC justify their choice of high-frequency rTMS with the fact that this brain-region has been related with processing of Theory of Mind challenges, that could thus be enhanced if cortical excitability of this area, and related networks, is increased (Enticott et al., 2011). Similarly, other isolated studies have chosen other targets according to the specific clinical outcome under study, generally choosing to apply high-frequency rTMS under the rationale that increasing cortical excitability in a particular area will result in improved performance of the function that these areas are believed to sustain (Fecteau et al., 2011; Enticott et al., 2012; Panerai et al., 2014). With only few exceptions (Sokhadze et al., 2009, 2010; Enticott et al., 2012; Panerai et al., 2014; Abujadi et al., 2017; Gómez et al., 2017) most studies also resort to stimulating cortical areas in both hemispheres, sometimes using complex stimulation schedules, where one hemisphere is stimulated first, then the other, and finally both in alternating succession. However, with the exception of the results reported by Enticott et al. (2012), the outcome of unilateral stimulation did not seem to be inferior to that of equivalent studies using bilateral targets.

Regarding stimulation protocols, there is also significant variability between different studies. A distinctive feature for most studies is the use of relatively conservative protocols, with stimulus intensities below resting motor threshold, the use of low frequencies within the range of high-frequency rTMS, and long intervals between successive TMS sessions. This could reflect fear of adverse effects, namely seizures, for which patients with ASD are at higher risk (Rossi et al., 2009). Predominance of such conservative protocols also makes results more difficult to interpret, as in these conditions the effects of rTMS are much more variable across individuals (Maeda et al., 2000).

### Limitations

Overall, interpretation of the existing literature with respect to the use of TMS for treatment of ASD is limited by concerns regarding study quality. In addition to the rare use of adequate control groups and control interventions, most studies did not safeguard against performance and detection bias, as neither subjects nor their caregivers were blinded with respect to treatment status, the same applying to outcome evaluators. The frequent use of parent- or caregiver-scored assessment scales, rather than direct assessments of clinical effects with the patients themselves, is also an important limitation and potential source of measurement bias. Moreover, while dropouts and exclusion of recruited individuals was a frequent occurrence, none of the reviewed studies adopted an intent-to-treat analysis.

In fact, the overwhelming majority of the reviewed studies showed one or both of two major methodological limitations: an inadequate control group and an inadequate follow-up period. For most dimensions of ASD core symptoms, a significant proportion of existing studies were non-controlled trials, and among controlled trials the vast majority compared outcome in the active rTMS arm with outcome in a waiting-list group. This means that placebo-effects, likely to be significant in an intervention like rTMS, cannot be ruled out, and are indeed a likely contributor for the observed effects. Only a very limited number of studies actually compared active rTMS to sham-rTMS (Fecteau et al., 2011; Enticott et al., 2012, 2014; Panerai et al., 2014; Ni et al., 2017), two of which (Fecteau et al., 2011; Ni et al., 2017) used a cross-over design, that is inferior to a classical sham-controlled design as subjects are more likely to discriminate between active and control interventions. Finally, several studies opted to compare rTMS treatment between patients with ASD and healthy individuals, which is not particularly informative for clinical practice. The other major limitation of most studies is the absence of an adequate follow-up period after the last stimulation. Only five studies assessed the stability of therapeutic gains 1 month or more after the end of treatment (Enticott et al., 2011, 2014; Panerai et al., 2014; Abujadi et al., 2017; Gómez et al., 2017), with the majority of studies only measuring therapeutic effects immediately after conclusion of rTMS. It is noteworthy that most of these limitations had already been noted by Oberman et al. (2016) in their review and, although we now have several more studies, the same limitations remain pertinent and prominent (Oberman et al., 2016). Furthermore, as of May 2018, a search of clinical trials.gov found few active or recruiting studies assessing the effects of TMS on various clinical symptoms dimensions of ASD. Of these, most were open-label single-group studies, or controlled but not blinded nor randomized, revealing an ominous trend to perpetuate the limitations of previous studies in the field.

Another unsettled issue is whether the effects of rTMS or iTBS on ASD symptoms vary with patient age, patient gender, or presence/absence of cognitive delay. Available studies, with the notable exception of Anninos et al. (2016), exclusively recruited adolescents or young adults, and most included a small number of female subjects in the active treatment arm with no separate reporting of the outcome measures for males and females. The same applies for the possible effects of co-morbid cognitive delay, with studies either excluding patients with cognitive delay or including a mix of subjects with and without cognitive delay (with the exception of Panerai et al., 2014, who only recruited patients with severe cognitive disability).

## Conclusions

In summary, there is limited preliminary evidence for a positive effect of rTMS on stereotyped and repetitive behavior, one of the core symptom dimensions of ASD, and even more limited evidence that rTMS has a positive effect on social behavior and some aspects of executive function. The significance of these findings is limited by concerns regarding the heterogeneity of data, as well as publication bias and the quality of the original studies. Critically, it remains unknown whether any such positive effects are lasting enough to be clinically useful. It is also unclear which stimulation parameters, targets and schedules offer the best opportunities for improvement or are most cost-effective. Moreover, it is unlikely that TMS will be able to achieve significant improvements in all symptom domains of ASD, with more limited effects likely to be found on more complex dimensions of behavior such as social behavior or Theory of Mind reasoning. Notwithstanding these limitations of the available studies, there seems to be reasonable evidence that clinical use of rTMS in patients with ASD is safe and well tolerated. In fact, there are no reports of serious adverse effects, and those that were reported were for the most part well tolerated and transient. Thus, there is a clear need for further, better-designed studies on the use of rTMS to treat ASD. To enhance the currently available data, future studies must be randomized, sham-controlled and double-blind, and should include adequate periods of follow-up after the end of treatment.

Our main, inevitable conclusion is that, at present, rTMS cannot be recommended as a viable or evidence-based treatment for ASD. Clear evidence of both short- and long-term efficacy is lacking, and it remains unclear whether the preliminary, limited evidence of a clinical effect is valid to all patient subgroups alike or which patient profile is most likely to benefit from this treatment.

## Author contributions

JB-C and AO-M planned and designed the study; AC, AV, and RL conducted the literature search and selected eligible studies; AC, AV, and JB-C extracted data; JB-C conducted data analysis; JB-C and AO-M wrote the manuscript that was reviewed by all authors.

### Conflict of interest statement

The authors declare that the research was conducted in the absence of any commercial or financial relationships that could be construed as a potential conflict of interest.

## Abbreviations

ASD: autism spectrum disorders
CI: confidence interval
cTBS: continuous theta burst stimulation
DLPFC: dorsolateral prefrontal cortex
DMPFC: dorsomedial prefrontal cortex
iTBS: intermittent theta burst stimulation
rTMS: repetitive transcranial magnetic stimulation
TMS: transcranial magnetic stimulation.
